# Selected Blood-Accessible Biomarkers in Prostate Cancer Radiotherapy: A PRISMA-ScR Timing-Window Framework Beyond PSA

**DOI:** 10.3390/cancers18152398

**Published:** 2026-07-25

**Authors:** Miloš Grujić, Barbara Alicja Jereczek-Fossa, Ivan Jovanović, Marija Živković Radojević, Giulia Marvaso, Katarina Krasić, Katarina Janković, Marija Peulić, Federico Mastroleo, Łukasz Kuncman, Vladan Mutavdžić, Milica Mihajlović, Neda Milosavljević

**Affiliations:** 1 Clinic of Radiation Oncology, University Clinical Center Kragujevac, 34000 Kragujevac, Serbia; marijazr@fmn.kg.ac.rs (M.Ž.R.); katarinakrasic@yahoo.com (K.K.); k.jankovic1991@gmail.com (K.J.); neda.milosavljevic@fmn.kg.ac.rs (N.M.); 2 Department of Clinical Oncology, Faculty of Medical Sciences, University of Kragujevac, 34000 Kragujevac, Serbia; 3 Department of Oncology and Hemato-Oncology, University of Milan, 20122 Milan, Italy; barbara.jereczek@ieo.it (B.A.J.-F.); giulia.marvaso@ieo.it (G.M.); federico.mastroleo@ieo.it (F.M.); 4 Department of Radiation Oncology, IEO European Institute of Oncology IRCCS, 20141 Milan, Italy; 5 Center for Molecular Medicine and Stem Cell Research, Faculty of Medical Sciences, University of Kragujevac, 34000 Kragujevac, Serbia; ivanjovanovic77@gmail.com; 6 Faculty of Medicine, University of East Sarajevo, 73300 Foca, Bosnia and Herzegovina; 7 Institute of Public Health, 34000 Kragujevac, Serbia; 8 Department of Oncology, Faculty of Medical Sciences, University of Kragujevac, 34000 Kragujevac, Serbia; marija.peulic@gmail.com (M.P.); vmutavdzic@yahoo.com (V.M.); 9 Center for Medical Oncology, University Clinical Center Kragujevac, 34000 Kragujevac, Serbia; 10 Department of Radiotherapy, Medical University of Lodz, 93-513 Lodz, Poland; lukasz.kuncman@umed.lodz.pl; 11 Department of External Beam Radiotherapy, Copernicus Memorial Hospital in Lodz Comprehensive Cancer Center and Traumatology, 93-513 Lodz, Poland; 12 Faculty of Medicine, University of Niš, 18108 Niš, Serbia; dr.milicaradic@gmail.com; 13 Clinic for Oncology, University Clinical Center Niš, 18108 Niš, Serbia

**Keywords:** prostate cancer, radiotherapy, biomarkers, γ-H2AX, interleukin-6, galectins, testosterone

## Abstract

**Background:** Blood-accessible biomarkers may support future personalization of prostate cancer radiotherapy, but their interpretation depends on sampling timing relative to radiotherapy, androgen deprivation therapy, and post-treatment recovery. We mapped clinical evidence for selected biomarker domains beyond prostate-specific antigen: γ-H2AX/DNA damage response, IL-6/inflammatory mediators, testosterone/endocrine recovery and a prespecified galectin-1/3 immune–stromal domain evaluated as a potential evidence gap. **Methods:** We conducted a PRISMA-ScR scoping review of PubMed, Scopus, and Web of Science searched on 7 January 2026. Eligible original human studies evaluated soluble serum/plasma analytes or peripheral blood cell-based assays in prostate RT pathways. Data were charted by biomarker domain, treatment context, RT modality/fractionation, assay reporting, sampling schedule, and endpoint linkage. **Results:** Of 3499 records, 45 studies were included. No eligible study reported repeated circulating galectin-1/3 kinetics anchored to prostate radiotherapy, identifying a distinct clinical evidence gap. The remaining evidence was dominated by testosterone studies (*n* = 28), followed by IL-6/inflammatory mediators (*n* = 12) and γ-H2AX/DDR (*n* = 5). Testosterone studies were mapped as separate RT-only endocrine kinetics and ADT-anchored recovery streams. IL-6 studies mainly used during-RT or early post-RT sampling and linked trajectories to acute toxicity, fatigue, symptoms or inflammatory phenotypes; no included study directly validated serial IL-6/inflammatory trajectories against biochemical control, metastasis-free survival, or overall survival. γ-H2AX studies were characterized by ultra-acute, fraction-anchored sampling. Across domains, baseline definition and sampling timing limited interpretability more than assay platform alone. **Conclusions:** Evidence maturity was unequal across the selected domains. Testosterone provided the comparatively more developed longitudinal clinical literature, whereas IL-6/inflammatory mediators remained exploratory and were linked mainly to acute toxicity, fatigue, symptoms, and inflammatory phenotypes. γ-H2AX remained predominantly a translational and biodosimetry-oriented marker, while galectin-1/3 represented a hypothesis-generating clinical evidence gap. None of these biomarkers currently supports routine biomarker-guided prostate RT personalization. Future biomarker-embedded studies may benefit from domain-specific sampling considerations, explicit RT/systemic-therapy context stratification, standardized assay reporting, and clinically relevant endpoints.

## Simple Summary

Blood tests may one day help doctors personalize radiotherapy for men with prostate cancer, but their usefulness depends on when blood samples are taken. In this scoping review, we examined clinical studies of four blood-accessible biomarker groups beyond prostate-specific antigen: markers of DNA damage, inflammation, immune–stromal biology, and testosterone recovery. We found that the available evidence is limited and uneven across the four biomarker groups. Testosterone and inflammatory markers have been studied most often, while studies of DNA damage markers have generally used very early sampling around radiotherapy fractions. No eligible studies measured repeated circulating galectin-1 or galectin-3 changes linked to prostate radiotherapy timing. None of these biomarkers currently supports routine personalization of prostate radiotherapy. The mapped evidence suggests that a single blood-sampling schedule may not be appropriate for all biomarkers. Future studies may therefore consider aligning sampling with each biomarker’s expected biological kinetics to better evaluate toxicity, recovery, and treatment response.

## 1. Introduction

Prostate cancer is the second most frequently diagnosed malignancy and the fifth leading cause of cancer-related mortality among men worldwide [1]. Radiotherapy (RT) is a cornerstone of curative treatment across risk groups, yet meaningful interpatient variability persists in tumor control, toxicity, and symptom burden despite similar clinical risk profiles and dose–volume constraints. Over the past decade, hypofractionated radiotherapy has become an evidence-based alternative to conventional fractionation for localized prostate cancer. Large randomized phase III trials of moderate hypofractionation, including CHHiP, PROFIT, and NRG Oncology RTOG 0415, demonstrated non-inferior disease-control outcomes compared with conventional fractionation, with long-term follow-up supporting the durability of this approach [2,3,4]. Mature randomized evidence also supports ultrahypofractionation. The 10-year HYPO-RT-PC analysis confirmed the long-term efficacy and safety of seven-fraction ultrahypofractionated radiotherapy in intermediate- to high-risk localized prostate cancer, while PACE-B demonstrated non-inferior 5-year freedom from biochemical or clinical failure with five-fraction SBRT in low- or intermediate-risk disease [5,6]. These findings are consistent with the low fractionation sensitivity (α/β) of prostate cancer suggested by radiobiological and meta-analytic evidence [7,8].

Current treatment personalization relies predominantly on clinicopathologic risk stratification and physical dosimetry, which do not directly capture biological heterogeneity in DNA damage response and repair capacity, inflammatory and immune signaling, stromal remodeling, or host endocrine milieu. This limitation is particularly relevant in hypofractionated RT, where higher dose per fraction may amplify interpatient differences in tissue response. Minimally invasive, blood-accessible biomarkers may eventually support biologically informed risk stratification, toxicity modeling, and dynamic monitoring during and after RT, but their clinical value depends on rigorous validation in appropriately designed prospective studies.

Among candidate blood-accessible biomarkers, γ-H2AX reflects radiation-induced DNA double-strand breaks and repair kinetics in peripheral blood cells [9,10]; IL-6 captures systemic inflammatory signaling related to tissue injury and stress responses and radiotherapy-associated inflammatory toxicity [11,12]; galectin-1/3 have been linked to prostate tumor–stroma biology and clinical outcome, radiation-associated immune regulation, and prostate-cancer radioresponse in preclinical models, while circulating Galectin-3 has also been explored in non-radiotherapy prostate-cancer cohorts [13,14,15,16,17]; and testosterone represents a clinically relevant endocrine axis in prostate cancer management and survivorship [18]. These domains were selected a priori not as an exhaustive biomarker panel and not as clinically equivalent evidence domains, but as complementary biological axes relevant to prostate radiotherapy: DNA damage response, inflammation, immune–stromal remodeling, and endocrine recovery. We anticipated that the evidence maturity would differ across domains. In particular, galectin-1/3 was retained as a prespecified immune–stromal domain because its biological rationale in radiation response, fibrosis-related biology, and tumor–microenvironment interactions made the presence or absence of radiotherapy-anchored circulating clinical evidence informative for gap mapping. The selection of these domains was intended to preserve biological coherence and to keep the synthesis centered on radiotherapy-anchored sampling kinetics rather than on the entire non-PSA biomarker landscape. Other blood-accessible and radiotherapy-relevant markers were therefore not treated as independent review domains. Routine inflammatory or hematologic indices, including C-reactive protein, neutrophil-to-lymphocyte ratio, and radiation-associated lymphopenia, were considered outside the primary scope unless reported within eligible IL-6/inflammatory mediator studies, because they represent broader systemic host-state or immune-cell-count phenotypes rather than a prespecified soluble or cell-based mechanistic domain. Similarly, TGF-β1 was charted when reported as part of eligible inflammatory mediator panels, but was not selected as a stand-alone domain. Functional radiosensitivity assays, such as radiation-induced lymphocyte apoptosis, were excluded when performed only as ex vivo assays without an in vivo circulating biomarker trajectory linked to prostate radiotherapy. Tumor-derived liquid-biopsy markers, including circulating tumor cells, cfDNA/ctDNA, microRNAs, and radiogenomic signatures, were not included as formal domains because they constitute large and methodologically distinct literatures focused mainly on tumor genomics, prognostic stratification, or inherited toxicity susceptibility rather than on repeated treatment-timed blood sampling around radiotherapy delivery [19,20,21,22]. These exclusions do not imply lack of clinical relevance; rather, they reflect the focused aim of this scoping review: to map selected blood-accessible biomarker domains in which timing of biospecimen collection is central to interpretation.

Previous syntheses have addressed overlapping components of this field but with different objectives. A prior systematic review and meta-analysis quantified the direction and magnitude of testosterone changes after prostate radiotherapy [18]. Broader reviews of prostate radiotherapy biomarkers have catalogued candidate markers of treatment response, normal-tissue radiosensitivity, and radiotoxicity, while liquid-biopsy and radiogenomic literature has focused primarily on tumor-derived nucleic acids, molecular prognostication, and inherited susceptibility to treatment-related toxicity [19,20,21,22,23,24]. These approaches are complementary to, but distinct from, the present review. We did not aim to re-estimate pooled biomarker effect sizes, comprehensively rank predictive performance, or review all genomic and liquid-biopsy platforms. Instead, our contribution is temporal and design-oriented: we map how biomarker sampling was anchored to radiotherapy fractions, radiotherapy completion, ADT exposure or cessation, and longitudinal recovery; distinguish RT-only testosterone kinetics from RT +ADT testosterone recovery; compare assay and endpoint reporting across selected domains; and identify where sampling design limits interpretation. This mapping provides the basis for an illustrative framework for future biomarker-embedded trial design rather than a recommendation for routine clinical use.

Therefore, we conducted a PRISMA-ScR-guided scoping review to map the extent, nature, and methodological characteristics of clinical studies evaluating selected circulating and blood-accessible biomarkers in prostate cancer radiotherapy pathways. This review is specifically organized around the temporal relationship between biomarker biology and radiotherapy delivery. Its central contribution is a timing-window framework for blood-accessible biomarkers in prostate radiotherapy: ultra-acute sampling for γ-H2AX/DNA damage response markers, during-treatment and early post-treatment sampling for IL-6 and inflammatory mediators, and longitudinal or ADT-anchored follow-up for testosterone. By mapping evidence across radiotherapy context, modality/fractionation, assay reporting, and endpoint linkage, we aim to describe differences in evidence depth and identify clinically important translational gaps, including the absence of RT-anchored circulating galectin-1/3 kinetics. This timing-focused synthesis is intended to inform the design of prospective biomarker-embedded studies in hypofractionated prostate radiotherapy, rather than to support immediate biomarker-guided treatment decisions.

## 2. Materials and Methods

### 2.1. Criteria for Inclusion and Exclusion

We conducted a scoping review of clinical studies evaluating circulating and blood-accessible biomarkers in men undergoing radiotherapy for prostate cancer. The review focused on four prespecified domains selected for biological relevance to radiotherapy response and feasibility for repeated peripheral blood sampling: γ-H2AX/DNA damage response, IL-6/inflammation, galectin-1/3 immune–stromal biology, and testosterone/endocrine recovery.

Eligible sources were original human studies, including prospective or retrospective cohorts, clinical trials, and translational studies embedded within prostate radiotherapy pathways. We included studies across radiotherapy modalities and fractionation regimens, including conventional EBRT, moderate hypofractionation, SBRT/ultrahypofractionation, brachytherapy, and particle therapy, as well as RT-alone and RT plus systemic-therapy contexts. RT + ADT studies were eligible when biomarker assessment was meaningfully linked to the prostate radiotherapy treatment pathway, including testosterone recovery anchored to ADT exposure or cessation. Studies were eligible when biomarker assessment was linked to radiotherapy delivery, follow-up, toxicity, symptoms, or clinically relevant endocrine recovery.

Studies including mixed treatment modalities, mixed radiotherapy sites, or broader clinical cohorts were eligible only when the prostate cancer subgroup, prostate radiotherapy pathway, or relevant biomarker data could be identified sufficiently for charting. Such studies were used only for the prostate radiotherapy-relevant information they contributed. Findings derived from non-prostate participants, non-radiotherapy treatment groups, or inseparable mixed cohorts were not extrapolated to prostate radiotherapy-specific clinical conclusions.

We excluded non-human studies, conference abstracts without full manuscripts, non-original publications, studies outside the prespecified biomarker domains, ex vivo-only radiosensitivity assays, dosimetry-only studies, and studies without meaningful linkage between biomarker reporting and the radiotherapy pathway.

Markers outside these prespecified domains, including stand-alone hematologic indices, routine inflammatory markers not embedded in eligible IL-6/inflammatory mediator studies, ex vivo-only radiosensitivity assays, circulating tumor cells, cfDNA/ctDNA, microRNAs, and radiogenomic signatures, were not included as independent domains because they fell outside the timing-window framework defined a priori for this review.

### 2.2. Sources of Information

We searched three bibliographic databases—PubMed, Scopus, and Web of Science—to capture clinical and translational sources of evidence at the intersection of prostate cancer radiotherapy and blood-accessible biomarkers. The final searches were run on 7 January 2026. The protocol was registered on the Open Science Framework (https://doi.org/10.17605/OSF.IO/WFPT5) on 22 December 2025, before the database searches were conducted on 7 January 2026. The protocol was updated on 15 February 2026 to clarify and broaden the radiotherapy context from a primarily hypofractionation-focused framing to prostate radiotherapy across fractionation regimens and to specify the eligibility of studies conducted within RT + ADT pathways, particularly testosterone-recovery studies anchored to ADT exposure or cessation. The update did not change the four prespecified biomarker domains, the databases searched, or the core concepts of the search strategy. All records screened before the protocol amendment were reassessed against the broadened eligibility criteria covering all radiotherapy fractionation regimens and eligible RT + ADT pathways. This reassessment did not change any title/abstract or full-text inclusion or exclusion decision and did not alter the final number or composition of included studies. Reporting follows the PRISMA extension for scoping reviews [25], and the completed PRISMA-ScR checklist is provided in Supplementary Appendix S2.

### 2.3. Strategy for Literature Search

The search strategy was designed around three concepts: prostate cancer, radiotherapy, and the prespecified biomarker domains (γ-H2AX, IL-6, galectins, and testosterone). The core query used across databases was: (“Prostatic Neoplasms” OR prostate cancer*) AND (“Radiotherapy” OR radiotherap* OR “external beam radiotherapy” OR IMRT OR irradiation) AND (“gamma-H2AX” OR “γ-H2AX” OR H2AX OR “DNA damage” OR interleukin-6 OR IL-6 OR galectin* OR testosterone).

The strategy was adapted as needed to match the syntax and indexing conventions of each database while preserving the same conceptual structure. In each database, we applied filters to restrict results to human studies and English-language records. Because this review was focused on prespecified biomarker domains, the search was not designed to capture the full prostate cancer liquid-biopsy literature, such as ctDNA, cfDNA, or microRNA studies, unless these overlapped with the selected domains. The full, database-specific search strategies are provided in the Supplementary Appendix S1.

Because the absence of eligible galectin-1/3 studies was a principal finding, we performed a post hoc sensitivity search on 11 July 2026 during revision to address gene-symbol and synonym sensitivity. The search was performed in PubMed, Scopus, and Web of Science using the same prostate cancer and radiotherapy concepts as the original search, with additional galectin-related terms including galectin-1, galectin-3, Gal-1, Gal-3, LGALS1, and LGALS3. Because this was a post hoc sensitivity search performed during revision, it was documented separately in Supplementary Appendix S1 and did not alter the main PRISMA flow diagram.

### 2.4. Process for Selecting Sources of Evidence

All retrieved records were imported into Zotero (version 7.0.30) for deduplication [26]. The deduplicated library was then uploaded to Rayyan to support screening and structured documentation of source-selection decisions [27].

Two reviewers (M.G. and N.M.) independently screened titles and abstracts against the predefined eligibility criteria. Records that were clearly outside the review scope, including non-prostate cancer studies, non-radiotherapy studies, records evaluating biomarkers outside the prespecified domains, non-human studies, or non-original publication types, were excluded at this stage. Full texts were obtained for all potentially eligible sources of evidence and for records in which eligibility could not be determined from the title and abstract alone. For reports not available through the indexed database record, additional retrieval attempts included searches of publisher websites, PubMed and Google Scholar, institutional library holdings, and alternative bibliographic records using the article title, authors, and publication details. Abstract-only conference records were also checked for a corresponding full-length publication. Author contact was not undertaken systematically.

**Figure 1:**
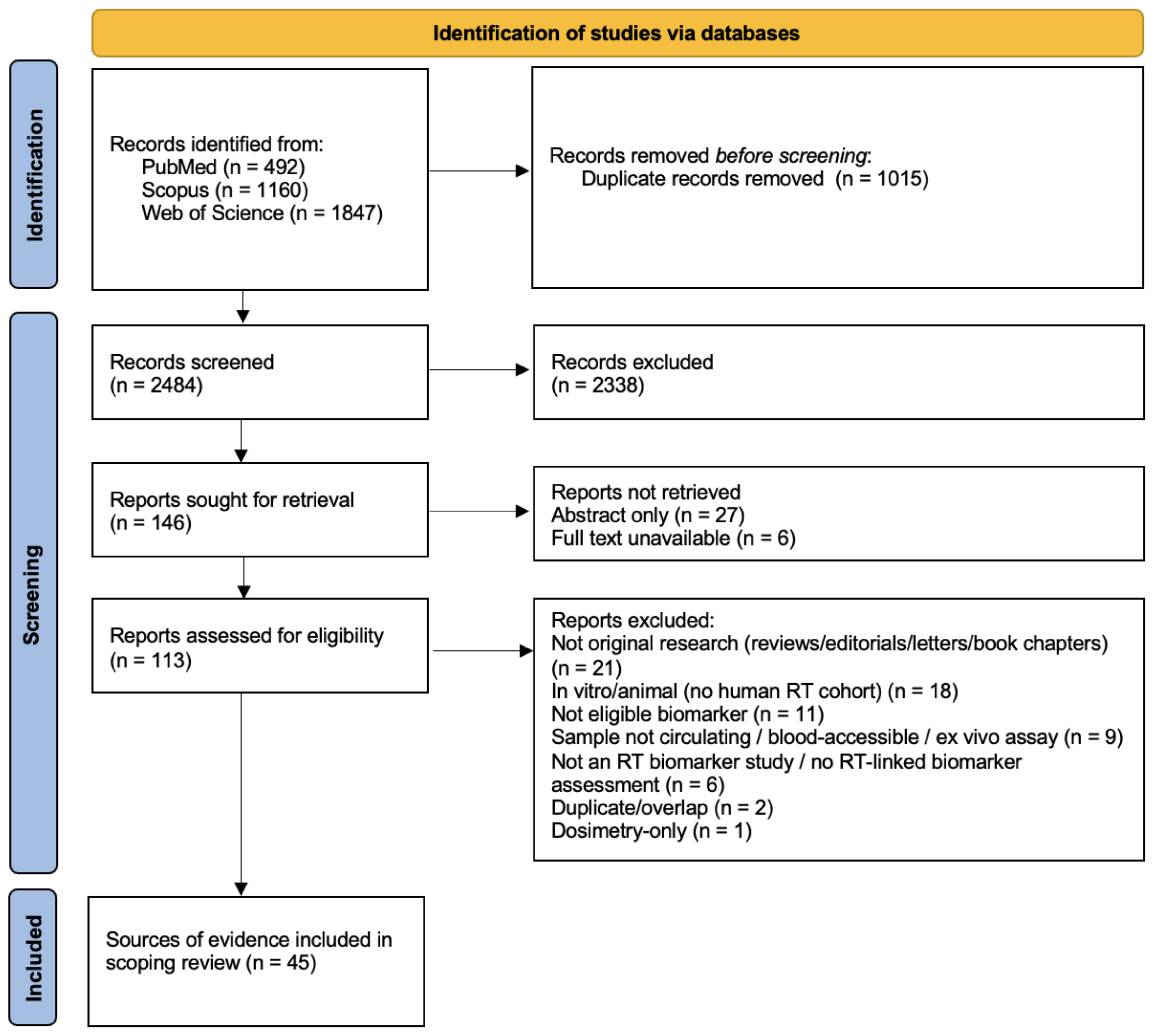
PRISMA flow diagram of source selection for the scoping review.

Full-text assessment was performed independently by the same two reviewers. Each excluded full-text report was assigned a single primary exclusion reason for transparent reporting. Disagreements were resolved by discussion, with a third reviewer (M.Ž.R.) available for adjudication. The selection process is summarized in the PRISMA flow diagram (Figure 1).

### 2.5. Data Charting Process and Data Items

Data were charted using a standardized a priori form capturing study characteristics, clinical and radiotherapy context, biomarker domain, specimen type, assay methodology, sampling schedule, and endpoint linkage. For each included study, we extracted publication details, design and sample size, patient setting, RT modality and dose–fractionation, systemic therapy context, biomarker(s) evaluated, blood specimen and assay platform when reported, sampling timepoints, and outcomes linked to biomarker behavior, including toxicity, symptoms, fatigue, biochemical control, or recovery trajectories. No single outcome hierarchy was imposed, consistent with the exploratory mapping purpose of a scoping review.

### 2.6. Critical Appraisal of Individual Sources of Evidence

A formal critical appraisal or risk-of-bias assessment was not performed. This decision was consistent with the objective of the scoping review, which was to map the extent, characteristics, and methodological design of the available sources of evidence rather than to estimate comparative treatment effects, pool associations, or make risk-of-bias-weighted conclusions.

### 2.7. Synthesis of Results

Findings were synthesized descriptively. Sources of evidence were grouped by biomarker domain and then mapped according to treatment context, radiotherapy modality/fractionation, specimen type, assay reporting, sampling schedule, and clinical or translational endpoint linkage. Particular emphasis was placed on the structure and timing of serial sampling, because the interpretability of circulating biomarkers depends on whether biospecimen collection is aligned with the expected biological kinetics of each biomarker domain.

To avoid implying equivalence across biomarker domains, we also prepared a descriptive evidence-position summary based on four mapped features: the volume of eligible evidence, the presence and structure of longitudinal sampling, linkage to clinical endpoints, and evidence of validation for clinical use. This summary was intended to describe the relative position of each domain within the mapped literature and did not constitute a formal quality assessment, certainty-of-evidence rating, or risk-of-bias appraisal.

## 3. Results

### 3.1. Study Selection

The database search identified 3499 records (PubMed *n* = 492; Scopus *n* = 1160; Web of Science *n* = 1847). After removing 1015 duplicates, 2484 records were screened and 2338 were excluded at title/abstract. We sought 146 full-text reports. Despite additional searches of publisher websites, bibliographic databases, Google Scholar, institutional library holdings, and corresponding full-publication records, 33 reports could not be retrieved: 27 were available only as conference abstracts, and the full text remained unavailable for six reports. Of 113 full-text reports assessed for eligibility, 68 were excluded: not original research, including reviews, editorials, letters, or book chapters (*n* = 21); in vitro or animal studies without a human prostate radiotherapy cohort (*n* = 18); biomarker not eligible (*n* = 11); sample not circulating/blood-accessible or ex vivo assay only (*n* = 9); not a radiotherapy biomarker study or no radiotherapy-linked biomarker assessment (*n* = 6); duplicate or overlapping report (*n* = 2); and dosimetry-only study without biomarker assessment (*n* = 1). Forty-five studies met the inclusion criteria (Figure 1). A post hoc galectin sensitivity search performed during revision is reported separately in Section 3.7 and Supplementary Appendix S1, because it did not identify additional eligible studies and did not alter the main PRISMA flow diagram.

### 3.2. Characteristics of Included Sources

The 45 included studies spanned multiple radiotherapy contexts, including RT alone, RT combined with androgen deprivation therapy (ADT), and RT delivered with other systemic approaches. The evidence encompassed contemporary and legacy treatment techniques, including conventionally fractionated EBRT/IMRT, moderate hypofractionation, SBRT/ultrahypofractionation, proton therapy, and brachytherapy. Table 1 provides a high-level study overview focused on biomarker domain, RT/systemic-therapy context, dominant sampling window, and principal clinical or translational endpoint linkage. Exact radiotherapy modality and dose-fractionation, specimen type, assay platform, and complete sampling schedules are provided in Supplementary Table S1.

A small number of studies involved mixed treatment modalities, mixed pelvic radiotherapy cohorts, or broader irradiation settings. These studies were retained only when the prostate cancer subgroup, prostate radiotherapy pathway, or relevant biomarker information could be identified sufficiently for charting. Their contribution was restricted to the extractable prostate radiotherapy-relevant methodological or biomarker information, and findings from inseparable non-prostate groups were not used to support prostate-specific toxicity, prognostic, or clinical-readiness conclusions. Studies without clear clinical endpoint linkage contributed to the mapping of biomarker kinetics, sampling design, or assay feasibility rather than to conclusions regarding clinical validity.

### 3.3. Overview of the Evidence Landscape for Biomarker Mapping

The 45 included studies were mapped by biomarker domain, treatment context, radiotherapy modality/fractionation, and sampling window. The evidence base was dominated by testosterone studies (*n* = 28), followed by IL-6/inflammatory mediator studies (*n* = 12) and γ-H2AX/DNA damage response (DDR) studies (*n* = 5). No eligible studies reported RT-anchored repeated circulating galectin-1/3 kinetics.

Across domains, heterogeneity was driven more by sampling design and baseline definition than by assay platform alone. Findings are therefore organized using prespecified kinetics windows: ultra-acute sampling for γ-H2AX/DDR, during-RT and early post-RT sampling for IL-6/inflammatory mediators, and longitudinal follow-up, including ADT-anchored recovery, for testosterone. Study-level characteristics are summarized in Table 1 and Supplementary Table S1, with cross-domain methodological reporting summarized in Supplementary Table S2.

The four domains were not equivalent in evidence maturity or clinical readiness (Table 2). Testosterone represented the comparatively more developed longitudinal clinical domain, although heterogeneous baseline definitions, recovery thresholds, and treatment contexts precluded its interpretation as a validated stand-alone radiotherapy decision marker. IL-6 and related inflammatory mediators constituted an exploratory clinical domain focused mainly on acute toxicity, fatigue, symptoms, and treatment-related inflammatory phenotypes, with limited linkage to late toxicity or oncologic outcomes. γ-H2AX/DDR remained predominantly a translational and biodosimetry-oriented domain, characterized by ultra-acute sampling and limited clinical endpoint validation. Galectin-1/3 represented a clinical evidence gap because no eligible study reported repeated circulating kinetics anchored to prostate radiotherapy delivery. None of the four domains currently supports routine biomarker-guided treatment selection or adaptation in prostate radiotherapy.

### 3.4. Testosterone Domain

Testosterone studies separated into two clinically distinct streams: RT-only endocrine kinetics and testosterone recovery after RT + ADT. These streams were mapped separately because their biological meaning, temporal anchor, and clinical relevance differ substantially.

RT-only cohorts included EBRT/IMRT, SBRT/ultrahypofractionation, proton therapy, and brachytherapy, with sampling typically extending from a pre-radiotherapy baseline through months-to-years follow-up. These studies most often evaluated testosterone decline, nadir, subsequent recovery and the occurrence of biochemical hypogonadism. Some also incorporated gonadal scatter-dose estimates or LH/FSH measurements to contextualize endocrine changes. Sampling was generally anchored to radiotherapy initiation or completion. Baseline definitions, sampling intervals, laboratory reference ranges, and integration with PROMs and dosimetry varied across studies.

In RT + ADT cohorts, sampling was generally anchored to ADT cessation and focused on time to recovery to prespecified testosterone thresholds. Studies frequently evaluated associations with ADT duration, age, baseline testosterone, nadir testosterone, or other clinical factors. Recovery definitions varied between laboratory-specific normal ranges and fixed biochemical thresholds, while measurement schedules ranged from protocolized trial follow-up to routine clinical intervals. Only a minority of studies paired testosterone recovery with symptom phenotypes or validated PROMs, whereas most reported recovery kinetics and, in selected studies, oncological outcomes.

### 3.5. IL-6 and Inflammatory Mediator Domain

The 12 included IL-6/inflammatory mediator studies were dominated by prospective cohorts with sampling during radiotherapy and early post-treatment. Timing frameworks varied from fraction- or dose-anchored schedules to broader week-based sampling, and studies used either single-analyte IL-6 measurement or multiplex inflammatory panels including TNF-α, TGF-β, IL-8, chemokines, and acute-phase reactants.

Clinical endpoint linkage was limited and the categories were not mutually exclusive. Four studies evaluated associations with acute toxicity, including acute GU/GI toxicity or proctitis [12,59,61,63]. One study evaluated late urinary and bowel toxicity [60], and two studies evaluated fatigue [56,61]. The remaining six studies focused primarily on cytokine kinetics, treatment-related trajectories, or correlative biomarker signals without direct evaluation of acute toxicity, late toxicity, fatigue, or oncological outcomes [11,57,58,62,64,65]. No included study directly evaluated serial IL-6 or related inflammatory trajectories as predictors of biochemical recurrence, metastasis-free survival, or overall survival. Although oncological outcomes may have been reported in some parent treatment studies, the biomarker analyses were not designed or reported to establish direct cytokine–oncological outcome associations [11,12,56,57,58,59,60,61,62,63,64,65].

Sampling schedules and baseline definitions varied across studies. In mixed RT + ADT cohorts, the timing of baseline sampling relative to ADT initiation was not consistently reported.

### 3.6. γ-H2AX and DNA Damage Response Biomarkers

γ-H2AX studies primarily evaluated ultra-acute DNA damage response signals in peripheral blood cells during radiotherapy, usually using paired sampling around the first fraction and immunofluorescence-based foci scoring. Most studies framed γ-H2AX as a biodosimetry or DNA damage response phenotype tool, exploring inter-individual variation in foci induction and decay. Sampling was typically concentrated in minutes-to-hours windows, with some studies including 24 h persistence or decay timepoints.

Across γ-H2AX studies, comparability was constrained by variation in blood processing, lymphocyte or leukocyte handling, fixation timing, scoring methodology, and reporting granularity of post-fraction timepoints.

### 3.7. Galectin Domain

No eligible studies evaluating circulating galectin-1/3 kinetics in relation to prostate radiotherapy delivery using repeat in vivo blood sampling were identified. The post hoc galectin sensitivity search conducted on 11 July 2026 identified 36 records; after deduplication, 28 records were screened, seven full texts were assessed, and no additional eligible clinical studies reporting repeated circulating galectin-1/3 kinetics anchored to prostate radiotherapy delivery were identified. The full sensitivity-search documentation is provided in Supplementary Appendix S1.

### 3.8. Cross-Domain Methodological Characteristics

Across biomarker domains, reporting varied in baseline definition, particularly in RT + ADT cohorts; biomarker sampling schedules; radiotherapy fields and dose-fractionation; assay and pre-analytical procedures; and integration of serial biomarker trajectories with standardized PROMs. Sampling patterns also differed across domains: γ-H2AX/DDR studies predominantly used minutes-to-hours intervals around radiotherapy exposure; IL-6/inflammatory mediator studies generally used during-RT and early post-RT assessments; and testosterone studies used longitudinal months-to-years follow-up. In RT + ADT studies, the temporal relationships among baseline sampling, ADT initiation, radiotherapy delivery, and ADT cessation were not consistently reported. Complete study-level methodological information is provided in Supplementary Tables S1 and S2.

## 4. Discussion

### 4.1. Principal Findings and What This Scoping Review Adds

**Figure 2:**
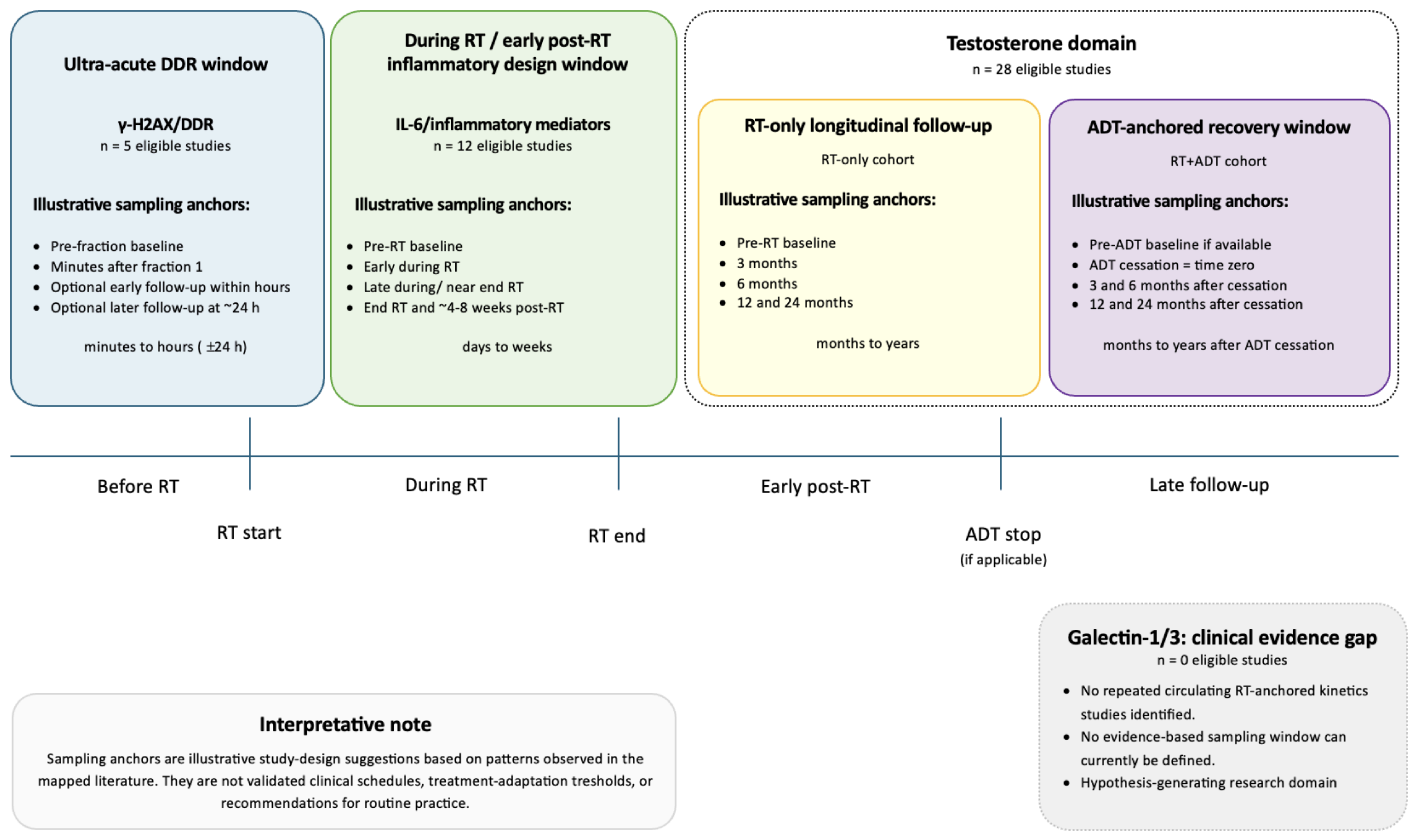
Illustrative timing-window framework for future blood-accessible biomarker studies in prostate radiotherapy. The figure summarizes the dominant sampling patterns identified in the mapped literature for γ-H2AX/DNA damage response biomarkers, IL-6/inflammatory mediators, and testosterone. RT-only testosterone kinetics and testosterone recovery after RT + ADT are presented separately because their biological interpretations and temporal anchors differ. Galectin-1/3 is visually separated and presented as a clinical evidence gap (*n* = 0 eligible studies), rather than as an established biomarker sampling window. All displayed sampling anchors are illustrative study-design considerations derived from patterns in the mapped literature and should not be interpreted as validated clinical schedules, treatment-adaptation thresholds, or recommendations for routine practice.

This PRISMA-ScR-guided scoping review mapped clinical evidence for circulating and blood-accessible biomarkers in prostate cancer radiotherapy and generated a timing-window framework to guide future biomarker-embedded study design (Figure 2). The principal finding is that biomarker interpretation depends not only on the selected analyte, but also on whether biospecimen collection is aligned with biomarker biology and treatment context.

Importantly, the four domains occupy distinct evidence positions and should not be interpreted as having comparable clinical readiness. Testosterone has the largest and comparatively more developed longitudinal clinical evidence base, although substantial heterogeneity remains in baseline definitions, recovery thresholds, treatment context, and endpoint linkage. IL-6 and related inflammatory mediators remain exploratory and are mainly associated with acute toxicity, fatigue, symptoms, and treatment-related inflammatory kinetics. γ-H2AX remains primarily a translational and biodosimetry-oriented marker with limited clinical validation, while galectin-1/3 represents a hypothesis-generating clinical evidence gap. Accordingly, the timing-window framework describes how future studies could be designed and should not be interpreted as supporting routine biomarker-guided treatment decisions.

Three findings are particularly relevant. First, the mapped evidence base was dominated by testosterone studies and IL-6/inflammatory mediator studies, whereas γ-H2AX/DNA damage response (DDR) studies formed a smaller but highly time-sensitive domain centered on ultra-acute fraction-anchored sampling; no eligible studies evaluated repeated circulating galectin-1/3 kinetics anchored to prostate radiotherapy delivery [11,12,28,29,30,31,32,33,34,35,39,40,41,42,43,44,45,46,47,48,49,50,51,52,53,54,55,56,57,58,59,60,61,62,63,64,65,66,67,68,69,70].

Second, across domains, heterogeneity in sampling timing and baseline definition appeared to limit interpretation more than assay platform alone. This was evident in inflammatory studies using variable week-, fraction-, or dose-milestone sampling during EBRT/IMRT [11,12,56,57,58,59,60,61,62,63,64,65], in testosterone studies where RT-only endocrine kinetics must be separated from ADT-anchored recovery [28,29,30,31,32,33,34,35,39,40,41,42,43,44,45,46,47,48,49,50,51,52,53,54,55], and in DDR studies where minute-to-hour sampling decisions are integral to interpreting induction and decay kinetics [66,67,68,69,70].

Third, no eligible study evaluated repeated circulating galectin-1/3 kinetics anchored to prostate radiotherapy delivery. Galectin-1/3 therefore represents a clinical evidence gap within the predefined review question. Mechanistic, tissue-based, and preclinical literature provides a hypothesis-generating rationale for prospective investigation but does not establish circulating clinical validity or readiness for implementation [14,15,71].

These findings are particularly relevant because hypofractionation is now a mainstream treatment standard in prostate cancer, supported by randomized evidence for moderate hypofractionation and ultrahypofractionated/SBRT approaches [5,6]. Yet current treatment personalization remains driven primarily by clinicopathologic risk groups and dosimetric parameters, which do not directly capture interpatient variability in DNA repair capacity, inflammatory response phenotypes, endocrine recovery, or symptom resilience. In this context, a timing-first evidence map provides practical design guidance for future biomarker-embedded hypofractionation studies, while emphasizing that current evidence remains insufficient for routine biomarker-guided treatment adaptation.

Relative to previous literature, the novelty of this scoping review lies primarily in the structure of the synthesis rather than in effect-size estimation or the identification of previously unreported biomarkers. The prior systematic review and meta-analysis of testosterone after prostate radiotherapy quantified aggregate endocrine changes [18], whereas our review maps the treatment context and temporal structure underlying those findings, including the distinction between RT-only endocrine kinetics and RT + ADT recovery, the definition of baseline and “time zero,” the density and duration of follow-up, recovery thresholds, and clinical endpoint linkage. Similarly, previous radiotoxicity biomarker, liquid-biopsy, and radiogenomic literature has primarily catalogued candidate markers or evaluated prognostic and predictive associations [19,20,21,22,23,24]. In contrast, the present review asks whether biospecimen collection was temporally aligned with biomarker biology and treatment exposure across several selected domains. Its principal added value is therefore a cross-domain temporal evidence map and an illustrative study-design scaffold for prospective biomarker-embedded trials, rather than a pooled estimate of biomarker effects or a comparative ranking of clinical performance.

### 4.2. γ-H2AX/DDR Biomarkers: Translational Potential and Time-Critical Feasibility

The γ-H2AX evidence identified in this scoping review was characterized by a relatively consistent methodological pattern: ultra-acute sampling anchored to radiotherapy exposure, immunofluorescence-based foci quantification in circulating blood cells, and interpretation as a readout of DNA damage signaling and early repair kinetics. Across included prostate radiotherapy studies, blood was typically collected immediately before treatment and within minutes after the first fraction, with additional timepoints capturing early decay or persistence, including standardized processing around 2 h or repeated sampling up to approximately 24 h [66,67,68,69]. This makes γ-H2AX the clearest example of a biomarker whose interpretability depends directly on fraction-anchored sampling. At present, however, γ-H2AX should be viewed as a translational and biodosimetry-oriented research tool rather than a clinically validated predictor of individual radiosensitivity in prostate radiotherapy [66,67,68,69].

Contextual evidence outside the mapped dataset supports the biological plausibility of time-resolved DDR phenotyping, but also illustrates the limits of immediate clinical implementation. In one prospective cohort, γ-H2AX foci decay ratios measured at 30 min and 24 h after ex vivo irradiation correlated with late toxicity grade and provided a threshold-based classifier for severe toxicity [10]. Conversely, studies comparing ex vivo γ-H2AX, apoptosis, and chromosomal sensitivity assays in prostate radiotherapy cohorts have reported more limited correlations with clinical radiosensitivity categories, emphasizing the need for standardized assay pipelines and external validation before clinical use [72].

Within the included DDR studies, informative signal was not simply initial γ-H2AX induction, but persistence and temporal patterning across early timepoints. The exploratory LDR brachytherapy study integrating γ-H2AX/53BP1 kinetics with longitudinal EPIC follow-up suggested that higher 24 h induction relative to baseline may correlate with later bowel symptom burden, whereas implant dosimetry alone showed limited correlation with late toxicity outcomes [70]. Although hypothesis-generating, this design illustrates how timing-resolved DDR signals may capture biological susceptibility not fully represented by dosimetric parameters.

The main barrier to translation is comparability. Even within this small evidence base, studies varied in blood handling, lymphocyte or leukocyte processing, fixation timing, scoring workflows, and reporting granularity of post-fraction sampling windows [66,67,68,69]. Future DDR studies in hypofractionated prostate radiotherapy may therefore consider standardized pre-analytical procedures and a minimal interpretable sampling set, potentially including baseline, approximately 30 min after fraction 1, and an early persistence or decay assessment at approximately 24 h, rather than expanding marker panels without equal attention to timing discipline [66,67,68,69].

### 4.3. IL-6 and Inflammatory Mediator Panels: Clinically Intuitive, but Highly Timing- and Context-Dependent

Across the included studies, IL-6 and related inflammatory mediators clustered predominantly within the during-RT and early post-RT window, with sampling designs ranging from dense longitudinal schedules to sparse milestone-based approaches. Several cohorts reported dynamic changes in circulating IL-6 during EBRT/IMRT and linked inflammatory trajectories to acute GU/GI toxicity, fatigue, or broader symptom phenotypes [11,12,56,59,60,61,62,63]. Studies using repeated within-treatment sampling were better positioned to capture transient or mid-course cytokine changes than designs relying mainly on baseline and end-of-treatment comparisons [12,56,57,60,63]. Within the mapped literature, IL-6 often increased during treatment, but the timing, magnitude, and detectability of this signal varied across cohorts; in some studies, other mediators or acute-phase reactants appeared more consistently detectable or more strongly associated with clinical subgroups [11,12,60,61,62,63].

The clinical endpoint scope of this evidence base was also narrow. Most included studies examined acute GU/GI toxicity, fatigue, symptom burden, or descriptive inflammatory kinetics, whereas direct associations with biochemical control, metastasis-free survival, or overall survival were not established [11,12,56,57,58,59,60,61,62,63,64,65]. This absence of direct biomarker–oncological outcome linkage should not be interpreted as evidence that IL-6 lacks prognostic relevance; rather, it indicates that such relevance remains largely untested in prospectively sampled prostate radiotherapy cohorts. Consequently, the current evidence supports IL-6 primarily as an exploratory treatment-response or toxicity-associated marker, not as a validated prognostic or treatment-selection biomarker.

Two interpretive constraints limit translation. First, baseline definition relative to systemic therapy was inconsistent. Studies comparing RT alone with RT plus androgen suppression, or tracking cytokines across pre-ADT, pre-RT, and post-RT phases, indicate that inflammatory mediator kinetics can be shaped by treatment context, complicating attribution of IL-6 changes to radiotherapy alone [57,58]. Second, variable timing anchors—week-based, fraction-anchored, or dose-milestone-based—make it unlikely that a single convenience schedule will capture the relevant inflammatory biology consistently [11,12,60,61,62,63]. Beyond treatment-phase timing, circulating IL-6 has important biological, pre-analytical, and analytical limitations. IL-6 is a pleiotropic and nonspecific marker of systemic inflammation, and within-patient concentrations may be influenced by transient inflammatory stimuli, time of day, acute infection, active inflammatory or metabolic comorbidity, recent strenuous exercise, and medications that modify inflammatory signaling. In prostate radiotherapy pathways, ADT introduces an additional treatment-related confounder because cytokine measurements obtained before ADT, after ADT initiation, and during radiotherapy do not represent equivalent biological baselines [57,58]. Biological variation is compounded by pre-analytical factors, including the choice of serum versus plasma, collection-tube type, the interval between collection and centrifugation, storage conditions, and freeze–thaw exposure. Assay-related factors are also relevant: absolute cytokine concentrations and limits of detection may differ across ELISA, multiplex bead-based, and electrochemiluminescence platforms, and measurements obtained using different platforms should not be assumed to be directly interchangeable [73,74,75,76]. These considerations are particularly important when IL-6 concentrations are close to the lower limit of quantification or when conclusions are based on a single measurement or an isolated treatment-related increase.

Consequently, current evidence does not support the use of IL-6 as a stand-alone decision-making biomarker in prostate radiotherapy. Future studies should use repeated within-patient measurements; standardize collection time, specimen matrix, processing interval, storage, and assay platform; analyze serial samples from the same patient within the same batch where feasible; and prospectively document ADT timing, acute infection, inflammatory comorbidities, and relevant concomitant medications. IL-6 may be more informative as one component of a prespecified multianalyte injury-response panel linked to clearly defined toxicity, fatigue, or patient-reported symptom phenotypes than as an isolated analyte interpreted against a universal threshold.

Mechanistic literature outside the mapped dataset further supports this interpretation. IL-6 signaling, particularly through the JAK/STAT3 axis, has been implicated in prostate cancer progression, treatment resistance, and radiotherapy response, including effects on apoptosis, stress signaling, and DNA repair-associated pathways such as ATM, ATR, and BRCA1/2 [77,78,79]. These data strengthen the rationale for monitoring IL-6 trajectories during prostate radiotherapy, but also reinforce the need for rigorous timing discipline and treatment-phase anchoring before IL-6 or inflammatory panels can be used for clinically interpretable risk stratification.

### 4.4. Testosterone: The Largest Longitudinal Evidence Base, but Clinical Meaning Depends on RT-Only vs. RT + ADT Context

The testosterone literature mapped in this scoping review separates into two clinically and biologically distinct streams: RT-only endocrine kinetics and testosterone recovery after androgen deprivation therapy (ADT) in combined-modality pathways. This distinction determines baseline definition, the appropriate “time zero,” and the clinical meaning of any trajectory. In RT-only cohorts, testosterone change is generally interpreted as a host endocrine effect plausibly related to scatter dose and/or perturbation of the hypothalamic–pituitary–gonadal axis. In RT + ADT cohorts, the central question is endocrine reconstitution after pharmacologic castration, with radiotherapy representing only one component of a broader treatment pathway [28,29,30,31,32,33,34,35,36,37,38,39,40,41,42,43,44,45,46,47,48,49,50,51,52,53,54,55]. Interpretation must therefore keep these streams separate to avoid misattributing ADT-driven kinetics to radiotherapy itself.

These findings complement rather than duplicate the previous systematic review and meta-analysis of testosterone changes after prostate radiotherapy [18]. Whereas that analysis estimated the overall direction and magnitude of testosterone change, the present scoping review focuses on the design heterogeneity underlying interpretation: RT-only versus RT + ADT context, the appropriate temporal anchor, baseline definition, sampling density, duration of follow-up, testosterone-recovery thresholds, and linkage to symptoms, quality of life, or oncological outcomes. These features are difficult to resolve through a pooled mean effect alone but are essential for designing future longitudinal biomarker studies.

Within RT-only cohorts, multiple series describe modest testosterone declines after EBRT/IMRT with variable recovery over approximately 12–24 months, often contextualized by pelvic exposure, field size, and inferred scatter dose [39,40,41,42,43,44,45,46,54]. Larger longitudinal datasets suggest that nadir commonly occurs within the first year and that some patients do not return to their own pre-RT baseline despite remaining within laboratory “normal” ranges, emphasizing within-person change rather than a single biochemical threshold [43,44,47]. Modality-specific evidence also supports a dose-distribution rationale: SBRT and LDR brachytherapy series generally report minimal to modest changes with recovery by approximately 18–24 months, often with patient-reported outcome data [49,50,51], whereas proton therapy series report little or no meaningful testosterone suppression in RT-only settings [52,53,55]. Overall, RT-only testosterone literature is comparatively more developed than the other mapped domains and may help frame expected endocrine trajectories in survivorship discussions; however, it does not validate testosterone as a stand-alone predictor of oncologic outcome, toxicity, or a basis for radiotherapy adaptation [43,44,45,47,50,55].

In RT + ADT pathways, the relevant anchor is usually ADT cessation rather than radiotherapy completion. Recovery kinetics are consistently influenced by ADT duration, age, baseline endocrine status, and nadir depth [28,29,30,31,32,33]. Randomized trial-derived datasets and pooled analyses show that longer ADT duration delays recovery and reduces the probability of returning to nonhypogonadal or laboratory-normal testosterone ranges [29,30], while analyses of castration depth suggest possible prognostic associations in selected settings [31]. Importantly, several studies link recovery trajectories with patient experience, including hot flashes and hormonal or sexual quality of life, connecting biochemical kinetics to survivorship burden in clinically meaningful ways [33,34,35].

### 4.5. Galectin-1/3: A Hypothesis-Generating Clinical Evidence Gap

Within the predefined scope of this review, no eligible study evaluated repeated circulating galectin-1 or galectin-3 kinetics anchored to prostate radiotherapy delivery. Accordingly, the mapped clinical literature provides no evidence regarding the optimal circulating specimen, treatment-related kinetics, sampling window, clinically relevant threshold, or association with toxicity, treatment response, or oncological outcomes. Galectin-1/3 should therefore not be interpreted as having evidence maturity comparable with testosterone, IL-6/inflammatory mediators, or γ-H2AX/DDR.

Contextual evidence outside the mapped clinical dataset provides only a hypothesis-generating rationale for future investigation. In human prostate tissue, increased stromal Galectin-1 expression has been associated with poorer clinical outcome, while prostate-specific preclinical work suggests that Galectin-3 inhibition may enhance radiation-induced cytotoxicity and radiosensitivity [14,15]. Separate clinical studies conducted outside radiotherapy-specific longitudinal pathways have demonstrated serum Galectin-3 detectability and reported associations with metastatic status or PSA-related clinical state [16,17]. These findings support the feasibility of circulating Galectin-3 measurement but do not establish radiotherapy-anchored kinetics, validated thresholds, predictive validity, or an evidence-based sampling window. More general radiotherapy literature has also linked Galectin-1 with radiation-associated immune suppression; however, such findings cannot be extrapolated directly to circulating biomarker performance in patients receiving prostate radiotherapy [13].

Prospective feasibility studies are therefore needed before a clinically meaningful galectin timing window can be proposed. Such studies should first establish circulating detectability, assay reproducibility, within-patient variation, treatment-related kinetics, and associations with predefined clinical endpoints. At present, galectin-1/3 should be regarded as a hypothesis-generating research domain rather than a clinically supported biomarker strategy.

### 4.6. Implications for Biomarker-Embedded Hypofractionation Trials

The principal practical contribution of this scoping review is not immediate clinical implementation, but design guidance for prospective biomarker-embedded hypofractionation trials. Across biomarker domains, the mapped evidence suggests that successful biomarker integration in prostate radiotherapy may depend less on expanding candidate panels than on considering whether sampling schedules are aligned with expected biomarker biology. γ-H2AX/DDR studies predominantly used ultra-acute, tightly standardized sampling around fraction delivery; IL-6 and broader inflammatory studies generally used structured during-treatment and early post-treatment assessments; and testosterone studies used longitudinal follow-up, with RT + ADT recovery analyses anchored to ADT cessation [11,12,28,29,30,31,32,33,34,35,39,40,41,42,43,44,45,46,47,48,49,50,51,52,53,54,55,66,67,68,69,70]. These observed patterns suggest that a uniform “one schedule fits all biomarkers” approach may be insufficiently informative. Figure 2 summarizes these patterns as an illustrative study-design scaffold. The displayed anchors are non-validated considerations for future research and should not be interpreted as clinical recommendations or validated schedules for treatment adaptation.

One possible future design could separate a universal clinical baseline from biomarker-specific sampling modules. Baseline assessment could be obtained before radiotherapy and, in RT + ADT pathways, before or clearly relative to ADT initiation. DDR sampling could include pre-fraction 1, approximately 30 min after fraction 1, and an early persistence or decay timepoint at approximately 24 h when feasible. Inflammatory mediator sampling could include pre-RT, early or mid-treatment, end-of-RT, and early post-RT assessments, for example at 4–8 weeks. Testosterone could be analyzed separately in RT-only and RT + ADT cohorts, with longitudinal assessment after RT in the former and recovery anchored to ADT cessation in the latter. Clinical and patient-reported endpoints could similarly be aligned with the biological window under investigation [12,34,35,46,56,60,61,63,70].

The potential clinical role of these biomarkers would likely be domain-specific rather than uniform across all patients. γ-H2AX/DDR assays could be evaluated in prospective studies of patients receiving high-dose-per-fraction regimens or those considered at increased risk of normal-tissue toxicity, where persistent DNA damage signals might ultimately support intensified surveillance or risk-adapted supportive care rather than immediate dose modification [10,70]. Serial IL-6/inflammatory mediator assessment may be more informative in patients undergoing pelvic radiotherapy or developing early treatment-related fatigue, urinary, or gastrointestinal symptoms, provided that ADT exposure, infection, inflammatory comorbidities, and concomitant medications are carefully accounted for [12,56,61,62,63,64]. Testosterone monitoring has the clearest potential relevance in survivorship after RT + ADT, particularly in older patients, those receiving longer-duration ADT, or those with low baseline testosterone or delayed endocrine recovery, where serial measurements may inform counseling, symptom assessment, and endocrine follow-up [28,29,30,31,32,33,34,35]. No clinically relevant patient subgroup can currently be defined for galectin-1/3 because RT-anchored circulating evidence is absent. These proposed use cases remain hypotheses for prospective validation and should not be used to guide routine treatment selection or adaptation.

Feasibility should be evaluated alongside biological validity. Repeated blood collection is generally less invasive than tissue-based biomarker assessment, but dense sampling schedules may increase patient burden, staffing requirements, phlebotomy coordination, specimen-tracking complexity, and the risk of missed protocol-defined windows. These considerations are particularly important for γ-H2AX, for which rapid post-fraction collection, tightly controlled processing intervals, immunofluorescence staining, microscopy, and foci scoring may be labor-intensive and difficult to integrate into routine radiotherapy workflows [66,67,68,69,70]. Automated or centralized image analysis may improve reproducibility and throughput, but would require technical validation and may not provide the turnaround time needed for real-time treatment adaptation. Multiplex inflammatory assays can reduce sample volume and evaluate several mediators simultaneously, but involve platform-specific detection limits, batch effects, quality-control requirements, and costs that may limit direct comparability across centers [75,76]. In contrast, testosterone measurement is widely available through routine clinical laboratory workflows, although repeated longitudinal testing still has cumulative resource implications. Future biomarker-embedded trials should therefore incorporate prospective feasibility metrics, including adherence to sampling windows, processing and reporting time, assay failure rates, personnel requirements, patient acceptability, and cost per evaluable longitudinal biomarker trajectory. A modular design, with a feasible core clinical sampling schedule and more resource-intensive translational substudies conducted at selected centers, may provide a more scalable approach than applying every assay at every timepoint.

The cost-effectiveness of these biomarker strategies remains unknown. None of the mapped evidence establishes that the added costs of serial sampling, specialized assays, and laboratory infrastructure are offset by improved toxicity prevention, treatment selection, quality of life, or oncological outcomes. Formal health-economic evaluation would therefore be premature at the current evidence stage. Prospective validation studies should first demonstrate analytical reliability and clinically meaningful incremental value, while collecting resource-use and cost data that could subsequently support cost-effectiveness analyses.

Several persistent gaps should shape future work. Many included studies were small, single-center cohorts or secondary analyses, with heterogeneous RT techniques, systemic therapy contexts, and endpoint definitions. Baseline definition was inconsistent, particularly in studies involving ADT, where endocrine and inflammatory measurements may reflect treatment-phase effects as much as radiotherapy effects [28,29,30,31,32,33,34,35,57,58]. Assay and pre-analytical reporting were also frequently incomplete, which is especially problematic for γ-H2AX studies, where processing and scoring pipelines are highly timing-sensitive [66,67,68,69,70], and for inflammatory markers, where host-state and treatment-context confounding may substantially influence signal interpretation [11,12,56,57,58,59,60,61,62,63]. Finally, no eligible RT-anchored circulating galectin-1/3 kinetics studies were identified in prostate cohorts. Although tissue-based and preclinical findings provide a biological rationale for investigation, they do not establish circulating biomarker feasibility, predictive validity, or an evidence-based sampling window in prostate radiotherapy [14,15,71]. Together, these observations support modular biomarker-embedded hypofractionation trials that integrate DNA damage response, inflammatory, immune–stromal, endocrine, and patient-reported outcome measures within a feasible clinical workflow.

Before any of these biomarkers can be considered for routine clinical use in prostate radiotherapy, they require prospective validation in clearly defined radiotherapy and systemic-treatment contexts. Translation will also require standardized specimen collection, processing, storage, and sampling procedures; assay harmonization and demonstration of inter-laboratory reproducibility; prespecified and externally validated thresholds or decision rules; and evidence that biomarker-guided use provides clinically meaningful benefit beyond established clinical and dosimetric factors. Feasibility, turnaround time, patient burden, resource use, and incremental costs should be recorded prospectively, followed by formal cost-effectiveness evaluation once analytical validity and clinical utility have been demonstrated. Until these requirements are met, the biomarkers reviewed here should remain investigational and should not be used for routine treatment selection or adaptation.

### 4.7. Limitations of the Present Scoping Review

Consistent with the aims of a scoping review, this study was designed to map the extent, characteristics, and methodological structure of the available evidence rather than to perform a formal risk-of-bias assessment, quantitative synthesis, or certainty-of-evidence grading. Associations reported in individual studies should therefore be interpreted as hypothesis-generating rather than definitive. Incomplete reporting of assay methodology, pre-analytical procedures, treatment context, and sampling schedules further limited direct comparison across studies. A small number of included studies involved mixed treatment modalities, mixed pelvic radiotherapy cohorts, or broader irradiation settings. Their contributions were restricted to extractable prostate radiotherapy–relevant biomarker, sampling, or methodological information; nevertheless, incomplete subgroup separation may have reduced the prostate-specificity and generalizability of some observations. Studies without clear clinical endpoint linkage informed the mapping of biomarker kinetics, sampling design, or assay feasibility but were not used to support conclusions regarding toxicity prediction, oncological prognosis, or clinical readiness.

The depth and maturity of the evidence were markedly unequal across the four prespecified domains. Testosterone accounted for most of the included studies and provided the largest longitudinal evidence base, whereas the IL-6/inflammatory and γ-H2AX/DDR domains were smaller and predominantly exploratory, and no eligible repeated circulating galectin-1/3 study was identified. Study counts should therefore not be interpreted as indicating equivalent evidential weight or clinical readiness across domains. In addition, no validated clinical thresholds, universally accepted reference changes, or treatment-adaptation cut-offs were identified for any of the mapped biomarkers. The available evidence is therefore insufficient to support biomarker-guided treatment selection or modification in routine prostate radiotherapy.

Clinical endpoint linkage was also limited. The IL-6/inflammatory mediator literature focused mainly on acute toxicity, fatigue, symptoms, and biomarker kinetics, with no direct validation against biochemical recurrence, metastasis-free survival, or overall survival. γ-H2AX studies were primarily biodosimetry- or translationally oriented, while testosterone studies more often addressed endocrine recovery and survivorship than validated prediction of radiotherapy efficacy or toxicity. Consequently, the clinical prognostic and predictive value of the mapped biomarker trajectories remains uncertain.

The search strategy was intentionally focused on prostate cancer, radiotherapy, and four selected biomarker domains and was not designed to capture the entire liquid-biopsy, radiogenomic, imaging, tissue, or multi-omic biomarker literature. Although the radiotherapy concept was broad, every modality-specific synonym was not explicitly enumerated, and some technique-focused reports may therefore have been less readily retrievable. The review was also restricted to English-language publications, and some potentially relevant reports could not be retrieved in full text.

Thirty-three potentially relevant reports could not be assessed in full, including 27 abstract-only records and six reports for which the full text remained unavailable despite additional retrieval attempts. Abstract-only records frequently lacked sufficient information on eligibility, assay methodology, treatment context, and sampling schedules. Nevertheless, exclusion of these reports introduces a risk of retrieval and availability bias and may have resulted in omission of eligible studies, particularly older studies or findings reported only at conferences. The completeness of the mapped evidence should therefore be interpreted with this limitation in mind.

A dedicated grey-literature search was not performed, and conference abstracts without full manuscripts were excluded. This may have limited the identification of ongoing, unpublished, negative, or incompletely reported studies. Furthermore, the published biomarker literature may preferentially represent statistically significant or positive associations. Because formal risk-of-bias, publication-bias, and selective-outcome-reporting assessments were outside the scope of this review, the extent of this possible reporting bias could not be quantified.

The original galectin search included the term “galectin*” but did not explicitly enumerate all gene-symbol variants, particularly LGALS1 and LGALS3. A post hoc sensitivity search using additional galectin synonyms and gene symbols was therefore performed in PubMed, Scopus, and Web of Science and identified no additional eligible studies. Although this reduced concern that the absence of eligible galectin evidence resulted solely from terminology sensitivity, the search was conducted during revision and did not constitute a complete updated search across all biomarker domains.

Finally, the mapped literature provided insufficient information on workflow feasibility, turnaround time, assay failure, personnel requirements, resource utilization, or cost-effectiveness. None of the included studies established that the additional costs and operational demands of serial sampling, rapid specimen processing, multiplex cytokine measurement, or γ-H2AX foci scoring were offset by improved toxicity prevention, quality of life, treatment selection, or oncological outcomes. The proposed timing-window framework should therefore be interpreted as illustrative study-design guidance requiring prospective analytical, clinical, operational, and economic validation rather than as an immediately scalable clinical pathway.

## 5. Conclusions

This scoping review mapped 45 studies across selected blood-accessible biomarker domains in prostate cancer radiotherapy and identified substantial differences in evidence depth, treatment context, and sampling design. Testosterone provided the largest longitudinal evidence base, but RT-only endocrine kinetics and testosterone recovery after RT + ADT were interpreted as separate evidence streams because their treatment-related temporal anchors differ. IL-6/inflammatory mediator studies remained predominantly exploratory and were linked mainly to acute toxicity, fatigue, symptoms, and treatment-related inflammatory trajectories. γ-H2AX/DDR studies were largely translational and biodosimetry-oriented, with interpretation dependent on ultra-acute, fraction-anchored sampling. No eligible study evaluated repeated circulating galectin-1/3 kinetics anchored to prostate radiotherapy, identifying a hypothesis-generating clinical evidence gap.

The principal contribution of this review is therefore design-oriented rather than practice-changing. Current evidence does not support routine biomarker-guided treatment selection, personalization, or adaptation in prostate radiotherapy. Considerations for future prospective biomarker-embedded studies include biomarker-specific sampling approaches, explicit definition of radiotherapy and systemic-treatment contexts, standardized pre-analytical and assay procedures, prespecified clinically relevant endpoints, and prospective evaluation of feasibility, reproducibility, resource use, and cost. External validation of biomarker thresholds and demonstration of incremental clinical utility will be required before implementation. Until these requirements are met, the biomarkers reviewed here should be regarded as tools for hypothesis generation and trial design rather than routine clinical decision-making.

## Figures and Tables

**Table 1:** Study-level overview of biomarker domain, treatment context, dominant sampling window, and principal clinical or translational linkage.

Study (Year)	Biomarker(s)	RT/Systemic-Therapy Context	Dominant Sampling Window	Principal Linkage
**Testosterone domain: RT + ADT or androgen-suppression contexts**				
Takei et al. [28] (2018)	Testosterone	RT + ADT	Post-ADT cessation; longitudinal months-to-years follow-up	Predictors of testosterone recovery
Nabid et al. [29] (2024)	Testosterone	RT + ADT	Post-ADT cessation; longitudinal follow-up	Recovery according to ADT duration
Ong et al. [30] (2025)	Testosterone	RT + ADT	Post-ADT cessation; longitudinal follow-up	Pooled testosterone-recovery kinetics
Zapatero et al. [31] (2021)	Testosterone/castration depth	RT + ADT	During ADT and subsequent follow-up	Castration depth and oncological outcomes
Murthy et al. [32] (2006)	Testosterone	RT + ADT	Post-ADT cessation; weeks-to-months follow-up	Testosterone-recovery kinetics
Padula et al. [33] (2002)	Testosterone	RT + ADT	Post-ADT cessation; months-to-years follow-up	Symptoms, QoL, and recovery
Dosani et al. [34] (2017)	Testosterone	RT + ADT	Post-ADT cessation; longitudinal follow-up	Hot flashes and testosterone recovery
Shah et al. [35] (2023)	Testosterone	RT + ADT	Post-ADT cessation; longitudinal follow-up	QoL and testosterone recovery
Bandara et al. [36] (2019)	Testosterone	RT + ADT	Baseline to 18–24 months	Fatigue phenotype
Pilepich et al. [37] (1989)	Testosterone	RT + ADT	During treatment	Treatment-exposure marker
Berg et al. [38] (2009)	Sex hormones, including testosterone	RT + CAB/antiandrogen	Baseline to 5-year follow-up	Long-term endocrine state and HRQoL
**Testosterone domain: RT-only and comparative contexts**				
Nichols et al., RTOG 9408 [39] (2017)	Testosterone	RT-only	End of RT to 3 months	Short-term testosterone kinetics
Zagars and Pollack [40] (1997)	Testosterone	RT-only	Baseline to 3 months	Kinetics and scatter-related exposure
Grigsby and Perez [41] (1986)	Testosterone with LH/FSH	RT-only	End of RT to 3–24 months	Endocrine-axis change and dosimetry
Tomić et al. [42] (1983)	Testosterone with LH/FSH	RT-only	One week to several months	Dose dependence and endocrine-axis change
Pickles et al. [43] (2002)	Testosterone	RT-only	Serial post-RT follow-up; months-to-years	Testosterone kinetics and biochemical control
Pompe et al. [44] (2017)	Testosterone	RT-only	Protocol-based longitudinal follow-up	Hypogonadism, kinetics, and biochemical control
Markovina et al. [45] (2014)	Testosterone	RT-only	Serial follow-up at approximately 6-month intervals	Biochemical hypogonadism
Ishiyama et al. [46] (2012)	Testosterone	RT-only plus gene-therapy context ^a^	During RT to long-term follow-up	Kinetics and scatter-dose association
Williams et al. [47] (2019)	Testosterone	RT-only	Long-term follow-up	Risk of iatrogenic hypogonadism
Usera et al. [48] (2020)	Testosterone	Mixed definitive-treatment cohort ^b^	Baseline prognostic assessment; separate no-ADT longitudinal subgroup	Pretreatment testosterone and survival outcomes
Oermann et al. [49] (2011)	Testosterone	RT-only	1–12 months	Kinetics and frequency of hypogonadism
Yuan et al. [50] (2019)	Testosterone	RT-only	3–24 months	Sexual QoL and testosterone kinetics
Taniguchi et al. [51] (2019)	Testosterone	RT-only	1–60 months, with dense early sampling	Testosterone nadir and recovery
Nichols et al. [52] (2012)	Testosterone	RT-only	End of RT to 24 months	Descriptive kinetics; no clear clinical endpoint
Kil et al. [53] (2013)	Testosterone	RT-only	End of RT to 12 months	Descriptive kinetics; no clear clinical endpoint
Planas et al. [54] (2016)	Testosterone with LH/FSH	RT-only	3 and 12 months	Endocrine-axis compensation
Hattori et al. [55] (2021)	Testosterone	RT-only versus RT + CAB	Months-to-years follow-up	Sexual function and QoL
**IL-6/inflammatory mediator domain**				
Stanojković et al. [12] (2020)	IL-6/inflammatory panel	RT-only	During RT; fraction-anchored	Acute toxicity
Lopes and Callera [11] (2012)	IL-6	RT-only	During RT; day-based sampling	During-RT cytokine kinetics
Holliday et al. [56] (2016)	Cytokine panel including IL-6	RT-only	During RT; fraction-anchored	Symptoms and fatigue
Johnke et al. [57] (2009)	Cytokine panel including IL-6	RT-only versus RT + androgen suppression	Pre-systemic therapy, pre-RT, and serial during-RT sampling	Cytokine kinetics and treatment-context confounding
Singh et al. [58] (2020)	Cytokine panel	RT + ADT	Pre-ADT to 3 months	Inflammatory trajectory across therapy
Christiansen et al. [59] (2007)	IL-6/TNF with hepcidin	RT-only	Pre-, mid-, and end-RT	Acute proctitis
Pinkawa et al. [60] (2015)	IL-6/TNF with CRP	RT-only	During RT to early post-RT	Late urinary and bowel toxicity
Kopčalić et al. [61] (2022)	IL-6 with TGF-β1	RT-only	Late during RT	Acute GU toxicity and fatigue
Bedini et al. [62] (2018)	Inflammatory mediator panel including IL-6	RT-only	Dose-milestone sampling during RT	Inflammatory mediator kinetics
Christensen et al. [63] (2009)	Cytokine panel including IL-6	RT-only	Serial during-RT sampling	Acute GU/GI toxicity
Hallemeier et al. [64] (2013)	IL-6 with TGF-β	Mixed pelvic RT cohort ^c^	Week 3 to final fraction	Descriptive kinetics; no clear clinical endpoint
Joensuu et al. [65] (2010)	Cytokine panel including IL-6	RT plus other systemic therapy	During RT	Correlative biomarker signal
**γ-H2AX/DNA damage response domain**				
Zwicker et al. [66] (2011)	γ-H2AX	RT-only	Minutes-to-hours around fraction 1	Biodosimetry and DDR
Zwicker et al. [67] (2015)	γ-H2AX	RT-only	Minutes-to-hours around fraction 1	Biodosimetry and DDR
Zahnreich et al. [68] (2015)	γ-H2AX with cytogenetic markers	Mixed RT contexts ^d^	Baseline, approximately 30 min, 24 h, and end of RT	DDR and biodosimetry
El-Saghire et al. [69] (2014)	γ-H2AX with blood transcriptomics	RT-only	Approximately 30 min after fraction 1 and 18–24 h later	DDR and immune signalling
Osman et al. [70] (2017)	γ-H2AX/53BP1	RT-only	Hours-to-months follow-up	Late toxicity and QoL signal

^a^ Ishiyama et al. evaluated testosterone kinetics in a no-ADT cohort treated with IMRT in the context of HSV thymidine kinase gene therapy; the study is therefore coded as RT-only + gene therapy context rather than pure RT-only. ^b^ Usera et al. included mixed definitive treatment modalities; a separate no-ADT longitudinal subgroup was used for testosterone trends. ^c^ Hallemeier et al. included mixed pelvic radiotherapy sites, with prostate cancer representing a subset of the cohort. ^d^ Zahnreich et al. included mixed radiotherapy contexts; prostate cancer formed the relevant partial-body radiotherapy subgroup. Abbreviations: ADT, androgen deprivation therapy; CAB, combined androgen blockade; CRP, C-reactive protein; DDR, DNA damage response; FSH, follicle-stimulating hormone; GI, gastrointestinal; GU, genitourinary; HRQoL, health-related quality of life; IL-6, interleukin-6; LH, luteinizing hormone; QoL, quality of life; RT, radiotherapy; TGF-β, transforming growth factor beta; TGF-β1, transforming growth factor beta 1; TNF, tumor necrosis factor; 53BP1, p53-binding protein 1; γ-H2AX, phosphorylated histone H2AX. Table note: Table 1 provides a high-level study map focused on biomarker domain, treatment context, dominant sampling window, and principal clinical or translational endpoint linkage. RT-only testosterone kinetics and testosterone recovery in RT + ADT pathways are presented as separate evidence streams because their biological interpretation, baseline definition, and temporal anchors differ. Sampling windows were harmonized to represent the dominant temporal design of each study rather than reproduce complete collection schedules. Exact radiotherapy modality and dose-fractionation, specimen type, assay platform, pre-analytical and processing details, and complete sampling timepoints are provided in Supplementary Table S1. No eligible study reported repeated circulating galectin-1/3 kinetics anchored to prostate radiotherapy; galectin-1/3 is therefore represented as a clinical evidence gap in the Results and Table 2 rather than as a study-level section in Table 1.

**Table 2:** Descriptive evidence position and current clinical interpretation of the prespecified biomarker domains.

Biomarker Domain	Eligible Studies	Descriptive Evidence Position	Dominant Linkage in the Mapped Evidence	Current Clinical Interpretation
Testosterone	28	Comparatively more developed longitudinal clinical evidence	RT-only endocrine kinetics; RT + ADT recovery; symptoms and quality of life in selected studies; oncologic outcomes in a minority	Useful for defining expected endocrine trajectories and future study design, but not validated as a stand-alone marker for RT selection or adaptation
IL-6/inflammatory mediators	12	Exploratory clinical evidence	Acute GU/GI toxicity, fatigue, symptoms, and inflammatory kinetics; no direct validation against biochemical recurrence, metastasis-free survival, or overall survival	Not validated as a stand-alone clinical decision-making biomarker
γ-H2AX/DDR	5	Translational and biodosimetry-oriented evidence	Ultra-acute DNA damage induction and decay; interindividual DDR phenotyping; limited clinical endpoint linkage	Research and biodosimetry tool without sufficient standardization or prospective clinical validation
Galectin-1/3	0	Clinical evidence gap; hypothesis-generating domain	No eligible repeated circulating RT-anchored kinetics studies	No current clinical readiness; prospective feasibility and validation evidence is currently lacking

Note: Evidence position was summarized descriptively according to the volume of eligible studies, longitudinal sampling structure, clinical endpoint linkage, and evidence of clinical validation. This classification is not a formal quality assessment, risk-of-bias evaluation, or certainty-of-evidence grading. ADT, androgen deprivation therapy; DDR, DNA damage response; GI, gastrointestinal; GU, genitourinary; RT, radiotherapy.

## Data Availability

All data generated or analyzed during this scoping review are included in this article and its Supplementary Materials. The review protocol was registered on the Open Science Framework: https://doi.org/10.17605/OSF.IO/WFPT5.

## Supplementary Materials

The following supporting information can be downloaded at: https://www.mdpi.com/article/10.3390/cancers18152398/s1. Supplementary Appendix S1: Full database-specific search strategies; Supplementary Appendix S2: PRISMA-ScR checklist; Supplementary Table S1: Study-level extraction for circulating and blood-accessible biomarkers in prostate cancer radiotherapy; Supplementary Table S2: Cross-domain methodological reporting and implementation overview of included studies.

## Abbreviations

The following abbreviations are used in this manuscript:

3D-CRT	Three-dimensional conformal radiotherapy
AA	Antiandrogen
ADT	Androgen deprivation therapy
ATM	Ataxia telangiectasia mutated
ATR	Ataxia telangiectasia and Rad3-related
BCR	Biochemical recurrence
BRCA1/2	Breast cancer gene 1/2
BT	Brachytherapy
CAB	Combined androgen blockade
cfDNA	Cell-free DNA
CFx	Conventional fractionation
CRP	C-reactive protein
ctDNA	Circulating tumor DNA
DDR	DNA damage response
EBRT	External beam radiotherapy
EPIC	Expanded Prostate Cancer Index Composite
FSH	Follicle-stimulating hormone
fx	Fraction(s)
GI	Gastrointestinal
GU	Genitourinary
h	Hour(s)
HRQoL	Health-related quality of life
HSV	Herpes simplex virus
IIEF-5	International Index of Erectile Function-5
IL-6	Interleukin-6
IMRT	Intensity-modulated radiotherapy
IPSS	International Prostate Symptom Score
JAK/STAT3	Janus kinase/signal transducer and activator of transcription 3
LDR-BT	Low-dose-rate brachytherapy
LH	Luteinizing hormone
miRNA	MicroRNA
mHypo	Moderate hypofractionation
OSF	Open Science Framework
PRISMA-ScR	Preferred Reporting Items for Systematic Reviews and Meta-Analyses extension for Scoping Reviews
PROMs	Patient-reported outcome measures
PSA	Prostate-specific antigen
QoL	Quality of life
RT	Radiotherapy
SBRT	Stereotactic body radiotherapy
SHIM	Sexual Health Inventory for Men
TAS	Total androgen suppression
TGF-β	Transforming growth factor beta
TGF-β1	Transforming growth factor beta 1
TNF	Tumor necrosis factor
TNF-α	Tumor necrosis factor alpha
UHfx	Ultrahypofractionation
VMAT	Volumetric modulated arc therapy
γ-H2AX	Phosphorylated histone H2AX
