# Supplementary material for: Selected Blood-Accessible Biomarkers in Prostate Cancer Radiotherapy: A PRISMA-ScR Timing-Window Framework Beyond PSA
Source: Cancers (Basel). 2026 Jul 25;18(15):2398. doi: 10.3390/cancers18152398 (PMC13463946; doi:10.3390/cancers18152398)
Supplement: Supplementary file 1 [file cancers-18-02398-s001.zip › Supplementary Appendix S1.pdf]

Supplementary Appendix S1. Full database-specific search strategies

The literature search was designed around three concepts: prostate cancer, radiotherapy, and the prespecified biomarker domains (γ-H2AX, IL-6, galectins, and testosterone). Final searches were run on 7 January 2026 in PubMed, Scopus, and Web of Science. Searches were restricted to English-language records. Non-human records were excluded using database filters or search terms where available and by manual screening, as described in the Methods.

1. PubMed

Search date: 7 January 2026
Search string:

```
(
  "Prostatic Neoplasms"[Mesh]
  OR prostate cancer*[tiab]
)
AND
(
  "Radiotherapy"[Mesh]
  OR radiotherap*[tiab]
  OR "external beam radiotherapy"[tiab]
  OR IMRT[tiab]
  OR irradiation[tiab]
)
AND
(
  "gamma-H2AX"[tiab]
  OR "γ-H2AX"[tiab]
  OR H2AX[tiab]
  OR "DNA damage"[tiab]
  OR interleukin-6[tiab]
  OR IL-6[tiab]
  OR galectin*[tiab]
  OR testosterone[tiab]
)
NOT
(
  animals[mh] NOT humans[mh]
```

)

Filters applied: English; Humans

2. Scopus
Search date: 7 January 2026
Search string:

TITLE-ABS-KEY (
("prostatic neoplasms" OR "prostate cancer*")
AND
("radiotherapy" OR radiotherap* OR "external beam radiotherapy" OR IMRT OR irradiation)
AND
("gamma-H2AX" OR "γ-H2AX" OR H2AX OR "DNA damage" OR "interleukin-6" OR IL-6 OR galectin* OR testosterone)
)
AND NOT TITLE-ABS-KEY (animal*)
AND NOT TITLE-ABS-KEY (mouse OR mice OR rat OR rats OR murine)

Filters applied: English language. Non-human records were reduced using animal-related exclusion terms and removed during screening when necessary.

3. Web of Science Core Collection
Search date: 7 January 2026
Search string:
TS=(
("prostatic neoplasms" OR "prostate cancer*")
AND
("radiotherapy" OR radiotherap* OR "external beam radiotherapy" OR IMRT OR irradiation)
AND
("gamma-H2AX" OR "γ-H2AX" OR H2AX OR "DNA damage" OR "interleukin-6" OR IL-6 OR galectin* OR testosterone)
)

Filters applied: English language. Non-human records were excluded during manual screening.

Notes on adaptation across databases

The search strategy was adapted to the syntax and indexing structure of each database while preserving the same conceptual framework. PubMed used MeSH terms where appropriate, whereas Scopus and Web of Science relied on title/abstract/keyword-topic searching. Non-human and clearly irrelevant records were removed through database filtering where possible and by title/abstract screening in accordance with the predefined eligibility criteria.

Post hoc galectin sensitivity search during revision

Because no eligible studies reporting repeated circulating galectin-1/3 kinetics anchored to prostate radiotherapy delivery were identified in the original search, a post hoc sensitivity search was performed during revision to address gene-symbol and synonym sensitivity. The search included additional galectin-related terms: galectin-1, galectin-3, Gal-1, Gal-3, LGALS1, and LGALS3.

Search date: 11 July 2026.

Databases searched: PubMed, Scopus, and Web of Science.

PubMed search string:

```
(
"Prostatic Neoplasms"[Mesh]
OR prostate cancer*[tiab]
OR prostate neoplasm*[tiab]
OR prostate carcinoma*[tiab]
)
AND
(
"Radiotherapy"[Mesh]
OR radiotherap*[tiab]
OR radiation[tiab]
OR irradiation[tiab]
OR "external beam radiotherapy"[tiab]
OR IMRT[tiab]
OR SBRT[tiab]
OR brachytherapy[tiab]
)
AND
(
galectin*[tiab]
OR galectin-1[tiab]
```

OR galectin-3[tiab]
OR Gal-1[tiab]
OR Gal-3[tiab]
OR LGALS1[tiab]
OR LGALS3[tiab]
)
NOT
(
animals[mh] NOT humans[mh]
)

Filters applied: English; Humans.

Scopus search string:

TITLE-ABS-KEY (
("prostatic neoplasms" OR "prostate cancer*" OR "prostate neoplasm*" OR "prostate carcinoma*")
AND
("radiotherapy" OR radiotherap* OR radiation OR irradiation OR "external beam radiotherapy" OR IMRT OR SBRT OR brachytherapy)
AND
(galectin* OR "galectin-1" OR "galectin-3" OR "Gal-1" OR "Gal-3" OR LGALS1 OR LGALS3)
)
AND NOT TITLE-ABS-KEY (animal* OR mouse OR mice OR rat OR rats OR murine)

Filters applied: English.

Web of Science search string:

TS=(
("prostatic neoplasms" OR "prostate cancer*" OR "prostate neoplasm*" OR "prostate carcinoma*")
AND
("radiotherapy" OR radiotherap* OR radiation OR irradiation OR "external beam radiotherapy" OR IMRT OR SBRT OR brachytherapy)
AND
(galectin* OR "galectin-1" OR "galectin-3" OR "Gal-1" OR "Gal-3" OR LGALS1 OR LGALS3)
)

Filters applied: English.

Records identified: 36
Records after deduplication: 28

Records excluded after title/abstract screening: 21
Full-text reports assessed: 7
Additional eligible studies identified: 0

The full-text reports assessed during the sensitivity search were excluded because they did not meet the eligibility criteria for this scoping review. Exclusion reasons included: no prostate radiotherapy cohort, tissue-only galectin assessment, preclinical/in vivo or in vitro design, non-prostate cancer model, conference abstract without full manuscript, or duplicate overlap with literature already identified as contextual mechanistic evidence. No study reported repeated circulating galectin-1 or galectin-3 kinetics anchored to prostate radiotherapy delivery.
