# Supplementary material for: Selected Blood-Accessible Biomarkers in Prostate Cancer Radiotherapy: A PRISMA-ScR Timing-Window Framework Beyond PSA
Source: Cancers (Basel). 2026 Jul 25;18(15):2398. doi: 10.3390/cancers18152398 (PMC13463946; doi:10.3390/cancers18152398)
Supplement: Supplementary file 1 [file cancers-18-02398-s001.zip › Supplementary Table S1.pdf]

Supplementary Table S1. Study-level extraction for serum/blood-accessible biomarkers in prostate cancer radiotherapy.

To ensure readability in Word, Table S1 is presented as three linked subtables (S1A–S1C) sharing a common Study ID.

Table S1A. Study and treatment characteristics

Study (Author–Year)	Country/setting	Design	N	Population/setting (definitive vs post-op; risk)	RT context (RT-only / RT+ADT / RT+Other)	ADT/other systemic details (agent; duration; timing)	RT modality (EBRT/IMRT/VMAT/SBRT/Proton/BT)	Dose–fractionation (exact)	Pelvic nodal RT (Y/N/NR)*
**Testosterone domain**									
Takei et al. [28] (2018)	Japan; Chiba University Graduate School of Medicine / Tokyo Shinjuku Medical Center	Retrospective cohort	82	Intermediate/high-risk PCa receiving EBRT + neoadjuvant ± adjuvant ADT (LHRH agonist ± bicalutamide)	RT+ADT	Intermediate-risk: neoadjuvant ADT 6 months; high-risk: neoadjuvant 6 months + adjuvant 2 years; very-high risk sometimes >3 years. LHRH agonist leuprorelin/goserelin; bicalutamide 80 mg daily in 76/82.	EBRT (3D-CRT)	72 Gy (3D-CRT)	NR
Nabid et al. [29] (2024)	Canada; multi-institutional (PCS III and PCS IV trials)	Secondary analysis of two multicenter phase III randomized trials with long-term follow-up (prospectively collected testosterone)	1230 randomized; 1092 included in testosterone recovery analysis (after exclusions)	Localized intermediate-risk (PCS III) and high-risk (PCS IV) PCa treated with RT with 0/6/18/36 months ADT	RT+ADT (with RT-alone reference group)	ADT: bicalutamide 50 mg for 1 month + goserelin acetate; durations 6, 18, or 36 months (exact duration enforced ±1 dose); RT-alone reference group	EBRT (pelvic + prostate for high-risk; prostate-only in some intermediate-risk)	IRPC: RT alone 76 Gy or RT+ADT with 70 or 76 Gy; HRPC: pelvic RT 44 Gy + prostate RT 70 Gy	Mixed (HRPC all pelvic RT; IRPC variable)
Ong et al. [30] (2025)	International MARCAP Consortium (5 randomized trials)	Individual patient data meta-analysis (pooled RCTs with prospectively collected serial testosterone)	1444 (from 1650 with testosterone recorded)	Definitive radiotherapy + GnRH-agonist–based ADT in intermediate/high-risk localized PCa; baseline noncastrate testosterone required	RT+ADT	Prescribed ADT duration cohorts: 4, 6, 18, 28, or 36 months; GnRH agonist ± oral antiandrogen; compliance data available	Definitive EBRT (dose variable by trial)	RT dose categorized <74 Gy vs ≥74 Gy (trial dependent)	NR
Zapatero et al. [31] (2021)	Spain; multicenter	Post hoc analysis of multicenter phase III randomized	154 (LTADT arm with ≥3 testosterone determinations during ADT)	Intermediate/high-risk localized PCa; high-dose RT + long-term ADT (28 months total)	RT+ADT	Goserelin: 2 months before RT + 2 months concurrent (STADT) then additional 24 months (LTADT); antiandrogen (flutamide 750 mg/d or bicalutamide 50 mg/d) first 2 months	High-dose EBRT (HRT)	≥76 Gy (range 76–82 Gy)	NR

		trial (DART01/05)							
Murthy et al. [32] (2006)	UK; Royal Marsden Hospital	Prospective sequential hormonal kinetics cohort	56 analyzable (59 enrolled; 3 excluded for insufficient timepoints)	Localized PCa T1c–T3a N0M0; radical prostate-only EBRT + short-course LHRHa	RT+ADT	Monthly depot goserelin 3.6 mg or leuprolide 3.75 mg for median 116 days (54–194); cyproterone 100 mg TID ×3 weeks to prevent flare; last injection scheduled week 1–2 of RT	CT-planned conformal EBRT (6–10 MV)	64–74 Gy to prostate ± seminal vesicles (nodes excluded)	N
Padula et al. [33] (2002)	USA; Memorial Sloan-Kettering Cancer Center	Prospective serial hormonal monitoring cohort	88	Localized PCa treated with neoadjuvant androgen deprivation (NAAD) + 3D-CRT; ADT stopped at end of RT	RT+ADT	Leuprolide acetate + bicalutamide; median NAAD duration 6 months (range 3–28); before and during RT; discontinued last day of RT	3D-CRT (external beam)	Median 75.6 Gy	NR
Dosani et al. [34] (2017)	Canada; BC Cancer Agency / University of British Columbia	Pre-specified secondary analysis of randomized phase III trial (ASCENDE-RT)	398 randomized; baseline testosterone available in 338; hot flash data in 392	Unfavourable-risk localized PCa receiving pelvic RT + boost (EBRT boost vs LDR-BT boost) plus 12 months ADT	RT+ADT	12 months LHRH agonist as 3-month depot injections (buserelin 9.56 mg or leuprolide 22.5 mg) + 4 weeks antiandrogen (flutamide 250 mg TID or bicalutamide 50 mg/d) at initiation; ADT cessation defined as last injection +3 months	Pelvic EBRT + boost: EBRT boost vs LDR-BT boost	Pelvic 46 Gy/23 fx then boost 32 Gy/16 fx (EBRT arm) or LDR-BT boost minimum peripheral dose 115 Gy	Y (pelvic irradiation 46 Gy/23 fx in all)
Shah et al. [35] (2023)	USA; single institution (MedStar Georgetown)	Retrospective review of prospectively collected institutional database	122	Localized PCa treated with short-course ADT followed by robotic SBRT (CyberKnife)	RT+ADT	Leuprolide for 3–6 months (77% received 3 months); SBRT typically delivered ~3 months after first injection	SBRT/UHfx (CyberKnife)	35–36.25 Gy in 5 fractions over ~2 weeks	N
Bandara et al. [36] (2019)	Australia; Calvary Mater Newcastle / University of Newcastle	Prospective cohort (registered clinical trial; serial PRO + blood biomarkers)	27	High-risk localized PCa receiving definitive EBRT + long-course ADT (18 months)	RT+ADT	Leuprorelin IM with 4 weeks antiandrogen at initiation; 6 months neoadjuvant then RT; total ADT 18 months	IMRT	Prostate 70 Gy at 2.5 Gy/fx; periprostatic 61.6 Gy; pelvic nodes 50.4 Gy (if indicated)	Mixed (pelvic nodes per nomogram risk)
Pilepich et al. [37] (1989)	USA/Canada/Puerto Rico; multi-institutional RTOG	Phase II randomized trial (RTOG 83-07)	197 analyzable (203 accrued)	Locally advanced adenocarcinoma (clinical stage B2/C; pelvic nodes allowed if below aortic bifurcation); definitive RT with hormonal cytoreduction	RT+ADT	Megestrol acetate 40 mg PO TID vs diethylstilbestrol (DES) 1 mg PO TID; started 2 months before RT and continued throughout RT	EBRT (regional lymphatics + prostate boost)	Regional lymphatics 44–46 Gy (up to 50 Gy acceptable) at 1.8–2.0 Gy/day + prostate boost 20–25 Gy at 1.8–2.0 Gy/day; total prostate 65–70 Gy	Y (regional lymphatics treated; periaortic included if pelvic nodes involved)

Berg et al. [38] (2009)	Norway; Norwegian Radium Hospital (SPCG-7 trial RT arm)	Cross-sectional HRQoL survey at 5 years with longitudinal sex hormone comparisons (within RCT cohort)	86 (respondents ; 97% response among eligible)	Locally advanced/high-risk localized PCa (mostly cT3) treated with definitive 3D-CRT + long-term nonsteroidal antiandrogen therapy	RT+ADT	Complete androgen blockade: leuprorelin 3.75 mg monthly + flutamide 250 mg TID for 3 months, then definitive RT + continuous flutamide; bicalutamide 150 mg daily if intolerance; antiandrogen ongoing at 5 years	3D-CRT (multileaf collimator)	70 Gy (1996–2000) or 74 Gy thereafter; 2 Gy/fx, 5 days/week; 15 MV photons	NR
Nichols et al., RTOG 9408 [39] (2017)	Multi-institutional NRG Oncology/RTOG	Secondary analysis of phase III randomized trial arm (NRG/RTOG 9408 RT-alone)	RT-alone randomized n=992; analyzable: baseline+end-RT T n=447; baseline+3mo T n=371	Localized PCa T1b–T2b, PSA <20; RT-alone arm; no hormonal therapy (excluding 2 off-protocol hormone starts)	RT-only	None (RT-alone arm; excluded patients who started hormones off protocol before evaluation)	EBRT (2D-EBRT/3D-CRT era)	WPRT 46.8 Gy + 19.8 Gy prostate boost = 66.6 Gy OR prostate-only 68.4 Gy; 1.8 Gy/fx	Mixed (most received WPRT; PORT for lowest-risk features)
Zagars and Pollack [40] (1997)	USA; MD Anderson Cancer Center	Retrospective cohort (subset randomly selected for paired hormone sampling)	85	Clinically localized PCa (T1–T3/T4, N0/NX, M0); definitive EBRT; no androgen ablation	RT-only	None (no androgen ablation)	EBRT (4-field box; some conformal 6-field boost)	46 Gy initial 4-field + boost to total 66–70 Gy (median 68 Gy) or 74–78 Gy (median 76 Gy); 2.0 Gy/fx daily	N
Grigsby and Perez [41] (1986)	USA; Mallinckrodt Institute of Radiology, Washington University (St Louis)	Prospective longitudinal cohort (endocrinologic sub-study)	59	Clinically localized-to-pelvis prostate carcinoma (Jewett A2–D1) treated definitively with EBRT; no prior hormonal therapy or curative operation	RT-only	None (explicitly no prior hormonal therapy)	EBRT (pelvic fields + prostate boost)	Whole pelvis 45 Gy in 25 fractions + prostate/adjacent boost 20–25 Gy (total 65–70 Gy); 20–35 MV linac; parallel-opposed pelvic fields + 270° anterior rotational arc boost	Y (entire pelvis treated to 45 Gy)
Tomić et al. [42] (1983)	Sweden; University of Umeå	Prospective + retrospective hormonal monitoring cohort	31 total; retrospective follow-up n=17; prospective paired pre/post n=14	Localized PCa treated with primary megavoltage EBRT	RT-only		EBRT (4-field box; 32.5 MV betatron)	58–71 Gy (mean 63.5 Gy); 2 Gy/day, 5 fx/wk over 8–10 weeks with 2–3 week break	NR
Pickles et al. [43] (2002)	Canada; British Columbia Cancer Agency	Prospective cohort registry	666	Radically irradiated prostate cases	RT-only	None (explicitly excluded any neoadjuvant/adjuvant androgen ablation)	Conformal EBRT (CT-planned)	Dose range 52.5–70 Gy (median 65 Gy); fraction	Mostly N (few pelvis/boo

	(Vancouver & Fraser Valley clinics)	analysis (BC Cancer Agency Prostate Cohort Outcomes Initiative)		since 1994; EBRT without neoadjuvant/adjuvant androgen ablation; baseline testosterone ≥5 nmol/L				size 1.6–2.6 Gy (most 2 Gy)	st cases ~2%)
Pompe et al. [44] (2017)	Canada; CHUM/University of Montreal (2002–2014; phase II/III trials)	Retrospective cohort using prospective trial data (multi-protocol; longitudinal testosterone)	248 assessable (from 425 treated; excluded ADT and missing data)	Localized PCa treated with definitive EBRT monotherapy (3D-CRT or IMRT; since 2007 mostly IMRT)	RT-only	None (neoadjuvant/adjuvant ADT excluded)	EBRT (3D-CRT/IMRT)	Total dose median 70 Gy (range 70–79); fraction counts varied by protocol (e.g., 39–41 fractions common)	NR (testis not shielded)
Markovina et al. [45] (2014)	USA; Washington University/Siteman Cancer Center (St Louis)	Prospective longitudinal cohort	51	Localized PCa treated with IMRT definitive (n=41) or post-prostatectomy fossa RT (n=10); excluded any hormone therapy within 12 months of any testosterone value; excluded high-risk NCCN	RT-only	None (no hormone therapy within 12 months of any testosterone measurement)	IMRT (5–8 fields; one Tomotherapy) ± LDR-BT boost in 4 patients	Definitive prostate median 73.8 Gy (7000–7920 cGy); post-op median 64.8 Gy (6480–6660 cGy). Hypofractionation subgroup 2.5 Gy/fx in 11 patients; 4 received 45 Gy EBRT + I-125 108 Gy boost; 1 received pelvic nodes 45 Gy.	Mixed (1 patient pelvic nodes 45 Gy)
Ishiyama et al. [46] (2012)	USA; Methodist Hospital/Baylor (phase I/II trial subset)	Prospective trial subset analysis (IMRT + gene therapy trial; subset without ADT)	29	Low-risk PCa; definitive IMRT + HSV-tk gene therapy; no ADT; no pelvic irradiation	RT+Other	HSV thymidine kinase gene therapy; no ADT; authors reported no significant effect on testosterone.	IMRT (NOMOS Peacock)	76 Gy in 35 fx (2 Gy/fx), 15 MV photons; prostate-only	N
Williams et al. [47] (2019)	USA; single Veterans Affairs institution (2002–2010)	Retrospective cohort (quality improvement)	149 (0 Gy control n=71; 45–54 Gy n=27; >60 Gy n=51)	PCa treated with EBRT, LDR-BT, or both; includes post-prostatectomy EBRT; ADT/TRT/chemo excluded	RT-only	Excluded any adjuvant/salvage ADT, orchiectomy, TRT, chemotherapy	EBRT ± LDR-BT (control = LDR alone)	EBRT groups: 45–54 Gy (with LDR boost) vs >60 Gy EBRT alone (max 79.2 Gy).	Mixed (65% of EBRT patients had pelvic node coverage)

Usera et al. [48] (2020)	USA; Huntsman Cancer Institute/University of Utah (institutional database)	Retrospective analysis of institutional outcomes database (prospectively gathered since 2012; retrospective before 2012) with propensity adjustment; plus subgroup longitudinal analysis (no ADT)	258 (outcomes cohort). Separate no-ADT subgroup used for testosterone trends (size not specified in excerpt)	Localized PCa across NCCN very-low to very-high risk; definitive therapy types incl EBRT, brachytherapy, EBRT+LDR, surgery; testosterone measured ≤100 days pre-therapy	Mixed (RT-only and RT+ADT both present; separate no-ADT subgroup analyzed for post-treatment T trends)	ADT captured as neoadjuvant/concurrent/adjuvant (details not specified in excerpt); separate subgroup never received ADT before/after definitive therapy for T trends	EBRT; Brachytherapy; EBRT+LDR (combined); Surgery	NR (therapy-type categories; no detailed dose/fx in excerpt)	NR
Oermann et al. [49] (2011)	USA; Georgetown University Hospital	Retrospective review of institutional protocol cohort	26	Low- to intermediate-risk localized PCa treated with SBRT monotherapy; ADT excluded	RT-only	None (ADT excluded)	SBRT/UHfx (CyberKnife)	36.25 Gy in 5 fractions (7.25 Gy/fx) over 2 weeks	N
Yuan et al. [50] (2019)	USA; UCLA/Georgetown/Genesis CyberKnife/VA network	Multi-institutional analysis of prospective SBRT trials (secondary analysis)	636	Low- and intermediate-risk localized PCa treated with prostate-only SBRT; no ADT	RT-only	None (no ADT)	SBRT/UHfx	Prostate-only SBRT: 35–36.25 Gy/5 fx, or 38 Gy/4 fx, or 40 Gy/5 fx	N
Taniguchi et al. [51] (2019)	Japan; Kansai Medical University	Retrospective cohort (single-center; serial labs)	102	Localized PCa treated with permanent interstitial LDR brachytherapy (I-125) monotherapy; no ADT	RT-only	None (explicitly no neoadjuvant or adjuvant ADT)	LDR brachytherapy (I-125 seeds)	Prescription dose 145 Gy (permanent implant)	N
Nichols et al. [52] (2012)	USA; University of Florida Proton Therapy Institute (Aug 2006–Oct 2007)	Prospective institutional protocol cohort analysis (PR01/PR02)	150 (of 171 enrolled; excluded 18 with ADT; pretreatment testosterone available for 150/153)	Low- and intermediate-risk localized PCa treated with definitive conformal proton therapy; no ADT; no pelvic nodal RT	RT-only	ADT excluded (17 before RT; 1 after RT excluded); no exogenous testosterone; no ADT started for failure during follow-up	Proton therapy (double-scattered)	78–82 CGE total; 2 CGE/fx; low-risk PR01: 78 CGE prostate; intermediate PR02: 46 CGE prostate+prox SV + boost 36 CGE to prostate (total 82 CGE if	N

								constraints allowed).	
Kil et al. [53] (2013)	USA; University of Florida Proton Therapy Institute	Prospective protocol cohort analysis	217 included (from 228 enrolled; 3 excluded for ADT/testosterone supplementation; 217 had baseline testosterone)	Low- and intermediate-risk localized PCa treated with hypofractionated passively scattered proton RT; no pelvic nodes; excluded ADT or testosterone supplementation	RT-only	Excluded ADT and testosterone supplementation prior to RT; no ADT given for failure during follow-up	Proton (passively scattered) hypofractionated	70–72.5 CGE at 2.5 CGE/fx (low-risk: 70 CGE prostate-only; intermediate-risk: 60 CGE prostate+prox SV + boost to 70 or 72.5 CGE)	N
Planas et al. [54] (2016)	Spain; Hospital Universitari Vall d'Hebron (Barcelona)	Retrospective comparative cohort (RP vs EBRT) with prospective blood draws	120 (RP 92; EBRT 28)	Clinically localized PCa (cT1–2). EBRT group all low-risk; RP group low/intermediate/high-risk mix.	RT-only (EBRT group explicitly without ADT)	None in EBRT cohort (explicitly no ADT before/during/after EBRT)	EBRT (IMRT to prostate+SV)	75 Gy total to prostate+SV, 2 Gy/day, 5 days/week (6 MV photons)	N (prostate+ SV only)
Hattori et al. [55] (2021)	Japan; Nagoya Proton Therapy Center (Nagoya City West Medical Center)	Prospective cohort subanalysis of IG proton therapy protocol with long-term follow-up	127	Localized PCa treated with image-guided proton therapy. Low-risk: IGPT alone. Intermediate/high/very-high: IGPT + neoadjuvant CAB (6–8 months) ± adjuvant CAB (high-risk total 24 months).	Mixed (RT-only in low-risk; RT+ADT in intermediate/high)	CAB: neoadjuvant 6–8 months pre-IGPT (intermediate/high); adjuvant 16–18 months post-IGPT recommended for high/very-high (total 24 months). Specific agents NR (CAB).	Proton therapy (image-guided; passive scattering and spot scanning)	Conventional fractionation: 2.0 Gy(RBE)/fx. Low-risk 74 Gy(RBE); intermediate/high 78 Gy(RBE).	N (prostate ± seminal vesicle portions; no nodes)
**IL-6 / inflammation domain**									
Stanojković et al. [12] (2020)	Serbia; Institute of Oncology and Radiology of Serbia (Jan 2016–Aug 2017)	Prospective longitudinal cohort	44	Localized/locally advanced PCa; definitive (n=21) or postoperative (n=23; 6 adjuvant, 17 salvage); no previous hormonal therapy	RT-only	No neoadjuvant or concomitant hormonal therapy (excluded)	3D-CRT	Definitive: 72 Gy/36 fx (2 Gy). Post-op: 66 Gy/33 fx (2 Gy).	Mixed (WPRT when LN risk ≥15%; post-op Nx/N1: pelvic nodes 44 Gy then boost)
Lopes and Callera [11] (2012)	Brazil; single center	Prospective single-	48	Prostate cancer treated with primary 3D-CRT	Mixed (RT-only and RT+ADT)	Hormone therapy present in 65% (details NR)	3D-CRT (6 MV photons)	74–78 Gy total; 2 Gy/fx daily	NR

		institution cohort		(some received concurrent hormone therapy)				(NCCN v1.2010-guided)	
Holliday et al. [56] (2016)	USA; MD Anderson/OHSU collaboration (Oregon Health & Science University)	Prospective longitudinal phase II study	28 enrolled (22 included in fatigue trajectory analysis)	Early-stage PCa receiving definitive EBRT; excluded concurrent ADT and brachytherapy; excluded baseline severe fatigue and major depression	RT-only	Excluded concurrent ADT	Definitive EBRT (technique not specified in excerpt)	39 fractions implied by cytokine sampling at 39th final fraction (exact total dose NR)	NR
Johnke et al. [57] (2009)	USA; East Carolina University (patients treated under RTOG 94-08/94-13 procedures)	Prospective cohort (serial plasma cytokines)	37 (xRT alone n=18; TAS+xRT n=19)	Stage I–II localized PCa receiving wide-field pelvic RT + prostate boost	Mixed (RT-only vs RT+ADT)	Total androgen suppression (TAS): flutamide 250 mg TID + goserelin (Zoladex) 3.6 mg SC q4w; started 2 months pre-RT and continued through RT (overall 112 days)	EBRT (4-field box; 20 MV)	Pelvic RT 46.8 Gy (RTOG 94-08) or 50.4 Gy (RTOG 94-13) at 1.8 Gy/fx, 5 fx/wk + prostate boost 19.8 Gy (total 66.6 or 70.2 Gy)	Y (wide-field pelvic regional lymphatics)
Singh et al. [58] (2020)	Australia; Royal Darwin Hospital / Alan Walker Cancer Care Centre	Prospective cohort (serial plasma cytokines)	18	Intermediate/high-risk localized PCa receiving neoadjuvant ADT followed by definitive IMRT (some continued adjuvant ADT up to 2 years)	RT+ADT	Neoadjuvant ADT with GnRH agonists/antagonists ± oral antiandrogens started 3–6 months pre-RT; 12 stopped after RT, 6 continued up to 2 years	IMRT (with fiducials and daily CBCT)	78 Gy/39 fx (n=4) or 80 Gy/40 fx (n=14)	NR (one patient had pelvic node involved prior to therapy)
Christiansen et al. [59] (2007)	Germany; University Hospital Göttingen	Prospective observational cohort (serial sampling)	18	Localized PCa (T1–T2 N0 M0) treated with primary curative 3D-CRT; endocrine therapy mentioned in PSA summary but not specified	Mixed/NR (primary RT; endocrine therapy status not clearly specified)	NR	3D-CRT (20 MV; Varian)	≥71 Gy to prostate (ICRU 50); daily fractions, 5×/week	NR
Pinkawa et al. [60] (2015)	Germany; RWTH Aachen University	Prospective observational cohort with biomarker sampling and QoL endpoints	91 (51 primary, 40 postoperative [23 adjuvant, 17 salvage])	Localized PCa treated with curative IMRT (definitive prostate RT or post-prostatectomy fossa RT)	Mixed (RT-only and RT+ADT)	Hormonal therapy per urologist (neoadjuvant/adjuvant in minority; ~14% primary, 25% post-op); agents NR	IMRT (5-field), 15 MeV photons; daily BAT ultrasound IGRT for definitive	2 Gy/fx. Definitive prostate 76 Gy; SIB to 18F-choline PET/CT-positive areas up to 80 Gy in most; Post-op fossa 66 Gy. Pelvic nodes	Mixed (20% pelvic nodes)

								(if indicated) 46 Gy.	
Kopčalić et al. [61] (2022)	Serbia; Institute for Oncology and Radiology of Serbia (Oct 2017–Feb 2020)	Prospective cohort	39	Localized/locally advanced PCa treated with definitive (n=26) or postoperative RT (n=13); exclusion: neoadjuvant/concomitant hormonal therapy and N1/M1	RT-only	Excluded neoadjuvant/concomitant hormonal therapy	3D-CRT (n=27) or VMAT (n=12)	Conventional 2 Gy/fx. Definitive: 72 Gy/36 fx. Post-op: 66 Gy/33 fx. Dose-volume groups: P only; P+SV; WPRT 44 Gy + boosts (as per institutional protocol).	Mixed (WPRT group present; definitive WPRT based on Roach formula; post-op Nx/N1 had pelvic nodes 44 Gy then boost)
Bedini et al. [62] (2018)	Italy; Fondazione IRCCS Istituto Nazionale dei Tumori, Milan	Prospective feasibility pilot cohort (serial plasma sampling)	20	Locally confined prostate adenocarcinoma treated with definitive IMRT 78 Gy; some received neoadjuvant/adjuvant hormones	Mixed (definitive IMRT; 6/20 received neoadjuvant/adjuvant hormone therapy)	Neoadjuvant/adjuvant hormone therapy in 6/20 (agents NR)	IMRT 78 Gy (IGRT referenced)	78 Gy, 2 Gy/fraction	NR
Christensen et al. [63] (2009)	Canada; Princess Margaret Hospital, Toronto (Dec 2006–Dec 2007)	Prospective longitudinal cohort (weekly sampling during IMRT)	42 (definitive n=22; post-prostatectomy n=20)	Intermediate-risk definitive IMRT (78 Gy/39) and post-prostatectomy IMRT (66 Gy/33); no hormone therapy	RT-only	None (explicitly no hormone therapy during study)	Image-guided IMRT	Definitive 78 Gy/39 fx; post-op 66 Gy/33 fx (2 Gy/fx)	NR (target volumes prostate vs fossa)
Hallemeier et al. [64] (2013)	USA; Mayo Clinic	Prospective observational pilot study	18 eligible (20 enrolled; 2 ineligible)	Pelvic RT patients for rectal (83%) and prostate (17%) cancers; pelvic RT 45–53.5 Gy; concurrent chemo allowed	Mixed (pelvic RT with/without chemotherapy; includes prostate cases)	NR (chemo yes/no recorded; ADT not detailed)	Pelvic RT: 3D-CRT (78%) or IMRT (22%)	Pelvis 45–53.5 Gy; daily 1.7–2.1 Gy, 4–5 days/week; boost allowed	Y (pelvic fields required by protocol)
Joensuu et al. [65] (2010)	Finland; single center (International Comprehensive Cancer Center Docrates / Helsinki	Phase I/II trial; nonrandomized single-center study	42	Nonmetastatic PCa, T2–T3N0M0; definitive RT; PSA <20; no neoadjuvant/adjuvant hormone therapy allowed	RT+Other	Gefitinib 250 mg orally daily; started 1 week before RT and continued throughout RT; hormone therapy not allowed	EBRT (3D-CRT)	50.4 Gy/28 fx to tumor, prostate and seminal vesicles, then 22 Gy/11 fx boost; total 72.4 Gy/39 fx	N

	University network)								
**γ-H2AX / DDR domain**									
Zwicker et al. [66] (2011)	Germany; University Hospital Heidelberg	Prospective comparative cohort (biological dosimetry study)	40 (20 3D; 20 SSIMRT)	Prostate RT patients; excluded prior RT and pelvic lymphatic irradiation	RT-only	NR	3D-CRT vs SSIMRT	3D: 72 Gy in 36 fx (2.0 Gy/fx); SSIMRT: 76 Gy in 35 fx (2.17 Gy/fx)	N
Zwicker et al. [67] (2015)	Germany; University of Heidelberg/DKFZ (2009–2010)	Prospective comparative cohort (quasi-randomized assignment in routine)	40 (20 SSIMRT; 20 TOMO)	PCa patients receiving prostate RT; excluded prior RT and pelvic lymphatic irradiation	RT-only	NR	SSIMRT vs Helical IMRT (Tomotherapy)	76 Gy cumulative; 2.17 Gy/fx; 6 MV photons	N
Zahnreich et al. [68] (2015)	Germany; University Medical Center Mainz	Prospective biodosimetry study during fractionated RT (partial- and total-body cohorts)	101 total; prostate cancer subgroup n=31 (partial-body RT)	Cancer patients including prostate cancer receiving partial-body RT (VMAT/RapidArc for prostate); subset had nodal irradiation; assessed DNA damage in peripheral leukocytes	RT-only / mixed systemic (chemo in other tumor types); ADT not specified for prostate	NR for prostate subgroup; breast/hematologic cohorts include chemo (not applicable to prostate)	Prostate: IMRT/VMAT (RapidArc) partial-body RT; all 6 MV photons	Fractionated RT with ~2 Gy per fraction (partial-body); 5 fractions/week; prostate specifics in supplement	Mixed (subset received lymph node RT)
El-Saghire et al. [69] (2014)	Belgium; Ghent University Hospital + SCK·CEN	Prospective translational paired-blood study around fraction 1	8	Prostate cancer patients receiving step-and-shoot IMRT	RT-only	NR	SSIMRT (Elekta Synergy)	2.09 Gy per fraction (tumor dose)	NR
Osman et al. [70] (2017)	UK (Northern Ireland); Queen's University Belfast / NI Cancer Centre	Prospective exploratory cohort (proof-of-concept)	12	Localized PCa treated with permanent LDR prostate brachytherapy (I-125 PPB) monotherapy	RT-only	None reported; multiple exclusions for inflammatory comorbidities/infection/diabetes	LDR brachytherapy (I-125 permanent implant)	145 Gy prescription	N

*Pelvic nodal RT coded as Y (pelvic nodes treated), N (prostate/prostate-bed only), or NR (not reported)

Abbreviations: ADT, androgen deprivation therapy; BT, brachytherapy; CAB, combined androgen blockade; CBCT, cone-beam computed tomography; CGE, cobalt gray equivalent; EBRT, external beam radiotherapy; fx, fractions; Gy, gray; IGRT, image-guided radiotherapy; IMRT, intensity-modulated radiotherapy; LDR-BT, low-dose-rate brachytherapy; LH, luteinizing hormone; LHRH, luteinizing hormone-releasing hormone; LN, lymph node(s); NR, not reported; PORT, prostate/prostate-bed only radiotherapy; SBRT, stereotactic body radiotherapy; SSIMRT, step and shoot intensity-modulated radiotherapy; VMAT, volumetric modulated arc therapy; WPRT, whole-pelvis radiotherapy.

Table S1B. Biomarker methods and sampling design

Study (Author–Year)	Biomarker domain	Biomarker(s) measured (exact)	Sample type (serum/plasma/whole blood)	Assay/platform (ELISA/CLIA/multiplex/IF foci/etc.)	Sampling schedule (standardized)	Dominant kinetics window (min–h / weeks / months / post-ADT stop)*
**Testosterone domain**						
Takei et al. [28] (2018)	Testosterone	Serum testosterone (baseline, nadir during ADT, serial after ADT termination)	Serum	Architect Testosterone II (Abbott)	Testosterone mainly every 3 months after ADT termination (some variable intervals)	post-ADT stop (m–y)
Nabid et al. [29] (2024)	Testosterone	Serum testosterone (serial)	Serum	NR (assay varies by center; normal ranges center-specific)	Baseline; q3 months for 18 months; q6 months to year 3; annually thereafter (to 21 years)	post-ADT stop (m–y)
Ong et al. [30] (2025)	Testosterone	Serial serum testosterone; recovery thresholds: noncastrate >1.7 nmol/L; nonhypogonadal >8.0 nmol/L; normal >10.5 nmol/L	Serum/plasma (trial labs; platform heterogeneous)	NR/variable across trials	Serial testosterone at prespecified trial time points; heterogeneity in exact dates across trials	post-ADT stop (m–y)
Zapatero et al. [31] (2021)	Testosterone	Serum testosterone (serial during ADT and follow-up); PSA at visits	Serum	Automated chemiluminescent immunoassay (centre laboratories; non-central)	Testosterone at every follow-up visit: q3mo year 1; q6mo to 5y; yearly thereafter; analysis used ≥3 during ADT; recovery defined >150 ng/dL after last 3-monthly injection	post-ADT stop (m–y)
Murthy et al. [32] (2006)	Testosterone	Serum testosterone, LH, FSH	Serum	Testosterone radioimmunoassay; LH/FSH enzyme assay	Baseline; during LHRHa; then 6, 12, 18, 24 and >40 weeks after last injection	post-ADT stop (m–y)
Padula et al. [33] (2002)	Testosterone	Serial serum testosterone; androgen-deprivation symptoms (weight gain, libido, breast symptoms, hot flashes, fatigue)	Serum	Radioimmunoassay (Diagnostic Product Corp); normal 270–1070 ng/dL; normalization ≥270 ng/dL	Months 1 and 4 post-treatment; then ~q6 months; recovery time from end of RT/ADT stop	post-ADT stop (m–y)
Dosani et al. [34] (2017)	Testosterone	Serum testosterone; patient-reported hot flashes frequency	Serum	NR (variable labs; acknowledged in limitations)	Testosterone: baseline; months 2,4,6,8,12,15,18,21,24; then q6 months. Hot flashes: q4 months to 1y, q6 months to 5y, then yearly; corresponding testosterone within 30 days.	post-ADT stop (m–y)
Shah et al. [35] (2023)	Testosterone	Total serum testosterone; EPIC-26 hormonal and sexual domain scores	Serum	NR	Testosterone at start of SBRT; q3mo for 1 year; q6mo thereafter; EPIC-26 pre-ADT, day 1 SBRT, and each follow-up	post-ADT stop (m–y)
Bandara et al. [36] (2019)	Testosterone	Serum testosterone; hemoglobin; fatigue PROMIS Fatigue-7a; MRI fat fraction and ADC (marrow)	Serum (testosterone), whole blood (Hb)	NR	Fatigue: baseline, 6, 12, 18 months<br>Testosterone/Hb: baseline, 3, 6,	post-ADT stop (m–y)

					9, 12, 18, 24 months<br>MRI FF/ADC: baseline and 6 months	
Pilepich et al. [37] (1989)	Testosterone	Serum testosterone (serial during treatment)	Serum	NR (not specified in excerpt)	Pre-hormones baseline; start of RT; end of RT (testosterone recorded throughout course)	weeks
Berg et al. [38] (2009)	Testosterone	Serum testosterone, estradiol, SHBG, LH, FSH; estimated free testosterone (testosterone×10/SHBG); PSA	Serum	Testosterone by Spectria coated tube assay; estradiol & SHBG by AutoDELFIA; LH/FSH by Access method	Baseline pre-treatment; 5-year follow-up blood draw (nonfasting, preferably 09:00–12:00)	months (3–24 m)
Nichols et al., RTOG 9408 [39] (2017)	Testosterone	Serum testosterone	Serum	Decentralized labs (no central standardization)	Enrollment baseline; RT completion; 3 months post-RT	weeks
Zagars and Pollack [40] (1997)	Testosterone	Total serum testosterone; PSA	Serum	Radioimmunoassay (Equate; Binax)	T0 pre-RT (within 1 month); T1 3 months post-RT	months (3–24 m)
Grigsby and Perez [41] (1986)	Testosterone	Serum testosterone, dihydrotestosterone, FSH, LH	Serum	Radioimmunoassay	Baseline pre-RT; completion of RT; 3, 6, 9, 12, 18, 24 months post-RT	months (3–24 m)
Tomić et al. [42] (1983)	Testosterone	Serum testosterone, LH, FSH, prolactin; testicular absorbed dose estimated	Serum	Radioimmunoassay	Prospective: baseline; 1 week post-RT; 3, 6, 12 months post-RT. Retrospective: single measurement 3–60 months (mean 20) post-RT.	months (3–24 m)
Pickles et al. [43] (2002)	Testosterone	Serial testosterone ratio to baseline; PSA; PSA doubling time in relapse	Serum	Radioimmunoassay	Baseline; then 3–6 monthly post-RT visits (1–2 m post-RT; 6 m; q6m to year 3; then yearly)	months (3–24 m)
Pompe et al. [44] (2017)	Testosterone	Serum baseline testosterone (BST) and follow-up testosterone ratios to BST	Serum	Clinical lab assays per trial protocol (platform NR)	Baseline testosterone, then follow-up according to individual trial protocols; testosterone follow-up not mandatory in all protocols; median 9 measurements per patient during follow-up	months (3–24 m)
Markovina et al. [45] (2014)	Testosterone	Total serum testosterone; biochemical hypogonadism status (≤300 ng/dL)	Serum	Clinical laboratory assay (reference 241–827 ng/dL); platform not specified	Baseline pre-RT; then q6 months for 2 years post-RT (6,12,18,24 m)	months (3–24 m)
Ishiyama et al. [46] (2012)	Testosterone	Total serum testosterone; PSA	Serum	Radioimmunoassay	Baseline; during week 6; post at 1.5, 4, 8, 12, 18, 24, 30, 36 m	months (3–24 m)
Williams et al. [47] (2019)	Testosterone	Total serum testosterone (routine with PSA)	Serum	Chemiluminescent immunoassay (Siemens Centaur System)	Baseline = average of all pre-RT values; follow-up mean T by year using ±6-month windows to 5 years	months (3–24 m)
Usera et al. [48] (2020)	Testosterone	Total serum testosterone (baseline); serial testosterone in no-ADT subgroup	Serum	Electro-chemiluminescent immunoassay	Baseline within 100 days pre-therapy; longitudinal post-therapy in no-ADT subgroup (evaluated at ≥1 year; serial mixed timepoints)	months (3–24 m)
Oermann et al. [49] (2011)	Testosterone	Total serum testosterone; PSA; PROMs (AUA/IPSS, SHIM, EPIC; SF-12)	Serum	NR	Baseline; 1 month post-RT; q3 months to 12 months (morning/early afternoon)	months (3–24 m)

Yuan et al. [50] (2019)	Testosterone	Serum testosterone; EPIC-26 sexual and hormonal domains	Serum	NR (draw times not standardized; diurnal variability acknowledged)	Follow-up windows: 3–6, 7–12, 13–18, 19–24 months post-SBRT (not fixed draws)	months (3–24 m)
Taniguchi et al. [51] (2019)	Testosterone	Serum total testosterone (STL); PSA	Serum	Chemiluminescent immunoassay on ARCHITECT i2000SR (Abbott)	Before BT; 1, 3, 6 months; then every 6 or 12 months to at least 24 months (up to 60 months)	months (3–24 m)
Nichols et al. [52] (2012)	Testosterone	Serum total testosterone	Serum	NR (not specified; decentralized clinical labs)	Baseline pre-RT; final week of RT; 6, 12, 18, 24 months post-RT	months (3–24 m)
Kil et al. [53] (2013)	Testosterone	Serum testosterone	Serum	NR	Baseline; final week of RT; 6 months; 12 months	months (3–24 m)
Planas et al. [54] (2016)	Testosterone	Serum LH, FSH, total testosterone, free testosterone, estradiol	Serum	Commercial immunoassays (specific platform NR); draws 07:00–10:00	Baseline (10–20 days pre-treatment) and 3 and 12 months after treatment completion	months (3–12 m)
Hattori et al. [55] (2021)	Testosterone	Serum testosterone and PSA (serial); sexual function: Erection Hardness Score and EPIC sexual domain (summary/function/bother)	Serum	NR	Testosterone/PSA: q3mo to 6 months, q6mo to 36 months, yearly thereafter; patient-reported EPIC/EHS: baseline pre-IGPT and 1,6,12,36,60 months post-IGPT	post-ADT stop (m–y)
**IL-6 / inflammation domain**						
Stanojković et al. [12] (2020)	IL-6/inflammation	Serum IL-1β, IL-2, IL-6, IFN-γ, TGF-β1; PBMC gene (IL-6, IFN-γ, TGF-β); immune cell subsets	Serum + PBMC/peripheral blood	ELISA (Invitrogen) for serum; RT-qPCR (TaqMan) for PBMC; flow cytometry	Serum: baseline; fx5; fx15; fx25; last fraction; 1 m post. PBMC gene: baseline and last fraction.	weeks
Lopes and Callera [11] (2012)	IL-6/inflammation	Serum IL-2, IL-4, IL-5, IL-6, TNF-α, MIP-1α, LIF; PSA (pre and 45 days post-RT)	Serum	Immunoassay kits: enzyme-amplified sensitivity immunoassay for IL-2/4/6/TNF-α/LIF; ELISA for IL-5 and MIP-1α (BioSource)	Cytokines: 1 day pre-RT; day 15 (≈30 Gy); day 30 (≈60 Gy). PSA: pre and 45 days post-RT.	weeks
Holliday et al. [56] (2016)	IL-6/inflammation	Serum IL-1α, IL-1β, TNF-α, IL-6, IL-8, IL-10, VEGF	Serum	Bead-based immunofluorescence assays (Millipore) on Luminex-100; duplicates	Baseline pre-fx1; 1 h post-fx5; 1 h post-fx15 (end wk3); 1 h post-fx25 (end wk5); 1 h post-fx39 (final)	weeks
Johnke et al. [57] (2009)	IL-6/inflammation	Plasma IL-1β, IL-6, TGFβ1	Platelet-free plasma	ELISA (Quantikine, R&D Systems)	xRT+TAS group: pre-TAS; post-TAS (immediately pre-RT); both groups: immediately pre-RT; 24 h after RT start; then during pelvic RT at cumulative doses 1.8, 9, 18, 27, 36, 45 Gy; during boost at 54 and 63 Gy	weeks
Singh et al. [58] (2020)	IL-6/inflammation	Plasma TNF-α, TGF-β1, IL-6, IL-8	Platelet-free plasma (lithium heparin tubes)	Sandwich ELISA (Life Technologies kits)	Before ADT; before RT; end of RT; 3 months post-RT	weeks
Christiansen et al. [59] (2007)	IL-6/inflammation	Plasma pro-hepcidin, ferritin, transferrin, IL-1β, IL-6, TNF-α; urine pro-hepcidin; CBC (Hb, WBC)	Plasma + urine (pro-hepcidin); whole blood (CBC)	ELISA kits (R&D for TNF-α; Hölzel for IL-6/IL-1β; DRG for pro-hepcidin); routine lab for CBC/iron markers	Plasma: before RT; mid-RT; end-RT. Urine (subset n=10): before RT and during RT (max). Toxicity scored weekly.	weeks

Pinkawa et al. [60] (2015)	IL-6/inflammation	Blood count (incl lymphocytes), acute phase proteins (CRP, ferritin, transferrin), cytokines IL-6 and TNF-α	Whole blood + serum/plasma (routine lab; cytokines reported as IL-6 and TNF-α)	Clinical laboratory assays; cytokines and acute phase proteins measured per hospital lab (platform NR)	T1 baseline pre-RT; T2 weeks 2–3; T3 weeks 4–5; T4 weeks 6–8; T5 6–8 weeks post-RT. QoL EPIC at baseline, last day, 6–8w post, and ~16 months.	weeks
Kopčalić et al. [61] (2022)	IL-6/inflammation	Serum IL-6 and TGF-β1 (Quantikine ELISA); fatigue (BFI); EPIC QoL; acute/late GU/GI toxicity (RTOG/EORTC modified by Peeters)	Serum	ELISA (R&D Systems Quantikine IL-6 and TGF-β1)	Serum cytokines: before RT and after 25th fraction. Toxicity graded every 5 fractions, end, 1 month post; late follow-up q3–6 months (max 30 months).	weeks
Bedini et al. [62] (2018)	IL-6/inflammation	Plasma IL-1β, IL-6, TNF-α; chemokines CCL2 and CXCL8; PTX3	Plasma (EDTA)	ELISA kits (R&D Systems)	Baseline; after 8 Gy; after 50 Gy; end of RT; 1 month post-RT	weeks
Christensen et al. [63] (2009)	IL-6/inflammation	Multiplex cytokines: GM-CSF, IFN-γ, TNF-α, IL-1α, IL-2, IL-6, IL-8, IL-10, IL-12p70 (plus urine/plasma pilot correlations)	Platelet-free serum (primary); serum/plasma/urine in pilot subset	Meso Scale Discovery Human Pro-inflammatory 9-Plex; SECTOR Imager 2400	Two baseline samples (education/CT-sim); then every 5th fraction (first 13 pts every 10th); plus last day; toxicity graded weekly (CTC-AE v3.0)	weeks
Hallemeier et al. [64] (2013)	IL-6/inflammation	Serum IL-6; plasma TGF-β	Serum (IL-6) + platelet-poor plasma (TGF-β)	Quantitative 2-site enzyme immunoassays (R&D Systems)	Pre-RT baseline; week 3 of RT; final week of RT	weeks
Joensuu et al. [65] (2010)	IL-6/inflammation	Serum TNF, IL-1α, IL-6 (correlative cytokines); tumor EGFR markers (tissue)	Serum	Cytometric bead array (BD Flex Set)	NR (correlative serum cytokine analysis performed; timepoints not specified in excerpt)	weeks
**γ-H2AX / DDR domain**						
Zwicker et al. [66] (2011)	γ-H2AX/DDR	γ-H2AX foci in lymphocytes (DSB biodosimetry); calibration curve from ex vivo irradiated volunteer blood	Peripheral blood lymphocytes/leukocytes (heparinized blood)	Immunofluorescence foci (anti-γ-H2AX, Upstate; DAPI); manual scoring	Blood pre-RT and 10 min after first fraction; leukocytes fixed 2 h post-irradiation; controls pre-RT	min–h (±24h)
Zwicker et al. [67] (2015)	γ-H2AX/DDR	γ-H2AX foci in lymphocyte nuclei	Peripheral blood lymphocytes/leukocytes (heparinized blood)	Immunofluorescence foci (anti-γ-H2AX) with manual counting (300 lymphocytes)	T0 pre-RT control; T1 10 min after first fraction; cells fixed 2 h after irradiation	min–h (±24h)
Zahnreich et al. [68] (2015)	γ-H2AX/DDR	γ-H2AX foci in peripheral leukocytes; cytogenetics (dicentrics + centric rings; acentric fragments)	Peripheral blood leukocytes/lymphocytes (Li-heparin)	γ-H2AX immunofluorescence foci (Upstate/Millipore) with Metafer imaging; plus cytogenetic scoring (Giemsa metaphases)	PBI patients: pre-RT; 30 min and 24 h after start of first fraction; and 30 min after start of last fraction	min–h (±24h)
El-Saghire et al. [69] (2014)	γ-H2AX/DDR	γ-H2AX foci in T-lymphocytes; whole genome expression (Affymetrix Human Gene 1.0 ST arrays) and qRT-PCR validation	Whole blood leukocytes/lymphocytes (heparin for γ-H2AX; EDTA for transcriptomics)	γ-H2AX immunofluorescence foci scoring (Cytovision); microarray + RT-PCR	γ-H2AX: before and 30 min after first fraction; transcriptomics: before first and second fraction (18–24 h after first fraction)	min–h (±24h)
Osman et al. [70] (2017)	γ-H2AX/DDR	γ-H2AX/53BP1 co-localized foci in peripheral blood lymphocytes; EPIC urinary/bowel/sexual domains	Peripheral blood lymphocytes (EDTA whole blood → Ficoll isolation)	Immunofluorescence foci staining for γH2AX (Ser139) and 53BP1; manual counting (200 cells/timepoint)	6 blood samples: pre-treatment; 1h, 4h, 24h post-implant; 4 weeks; 3 months. EPIC at baseline, 1m, 3m, 6m, 9m, 1y, 2y, 3y.	min–h (±24h)

[*]Kinetics windows: ultra-acute (min–h, ±24 h), during-RT/early post-RT (weeks), longitudinal follow-up (months–years), and recovery anchored to ADT cessation (post-ADT stop).

Abbreviations: CBC, complete blood count; CLIA, chemiluminescent immunoassay; CRP, C-reactive protein; DAPI, 4′,6-diamidino-2-phenylindole; DDR, DNA damage response; DSB, DNA double-strand break; EDTA, ethylenediaminetetraacetic acid; ELISA, enzyme-linked immunosorbent assay; h, hours; IF, immunofluorescence; IL, interleukin; IL-6, interleukin-6; m, months; min, minutes; NR, not reported; PBMC, peripheral blood mononuclear cells; qPCR, quantitative polymerase chain reaction; RIA, radioimmunoassay; RT-qPCR, reverse transcription quantitative polymerase chain reaction; TGF-β1, transforming growth factor beta 1; TNF-α, tumor necrosis factor alpha; wk, weeks; y, years; γ-H2AX, phosphorylated histone H2AX.

Table S1C. Endpoints and key findings

Study (Author–Year)	Endpoints assessed (tox/QoL/oncologic/biodosimetry)	Key finding (1 line)	Notes (baseline definition; confounders, and implementation consideration)
**Testosterone domain**			
Takei et al. [28] (2018)	Time to recovery to supracastrate (>50 ng/dL) and normal (>200 ng/dL) testosterone; predictors (ADT duration, nadir T, 6-month T)	ADT duration ≥33 months and nadir testosterone ≤20 ng/dL predicted delayed recovery to supracastrate/normal levels; testosterone >50 ng/dL at 6 months predicted recovery to normal.	Retrospective; measurement intervals variable; RT technique 3DCRT only.
Nabid et al. [29] (2024)	Rate and time to recovery to normal testosterone; predictors (age/comorbidities/ADT duration)	Recovery to normal testosterone decreased with longer ADT (0/6/18/36 mo: 87.4%/75.9%/54.8%/43.2%); median time to recovery among those who recovered increased from 0.31 to 5.0 years with longer ADT.	Recovery defined as return to center-specific normal range; analysis censored at biochemical recurrence to avoid re-ADT effect.
Ong et al. [30] (2025)	Time to testosterone recovery; nomograms; association between effective castration period and metastasis-free survival	Testosterone recovery varies strongly by ADT duration, baseline testosterone and age; effective castration period showed nonlinear association with MFS (notably for 6-month ADT).	High-level prospective testosterone dataset; not an RT biomarker-to-toxicity study but essential for TRT/recovery kinetics in RT+ADT context.
Zapatero et al. [31] (2021)	bDFS, MFS, OS; time to testosterone recovery	Median testosterone ≥50 ng/dL during ADT associated with worse bDFS; deeper suppression <20 vs 20–49 ng/dL not associated with better outcomes; median time to recovery 16.3 months (IQR 1.7–70.9).	Testosterone not centrally measured; recovery threshold >150 ng/dL based on lab lower limit; analysis restricted to LTADT arm with serial TT.
Murthy et al. [32] (2006)	Time to testosterone recovery (>6 nmol/L); predictors using Cox model	No men normal at 6 weeks; 35% normal at 12 weeks; 85% at 18 weeks; 89% at 24 weeks; 96% by 1 year; median recovery 13 weeks.	Monthly depot only (no 3-month); assay change corrected via regression; baseline LH/FSH predicted recovery.
Padula et al. [33] (2002)	Time to testosterone normalization; ADT-related symptoms at 1-year landmark	65% normalized; median time to normalization 18.3 months; normalization rates 10/26/38/59% at 3/6/12/24 months; low-normal baseline testosterone predicted delayed normalization; symptoms at 1 year strongly associated with non-normalization.	Pretreatment testosterone recorded in 39%; duration of NAAD not predictor; recovery continued up to 72 months.
Dosani et al. [34] (2017)	Time to testosterone recovery (thresholds 5/7.5/10 nmol/L); hot flash onset/peak/cessation relative to testosterone	Hot flashes began/peaked at ~4 months from first injection at castrate testosterone; median cessation 7.6 months after ADT cessation at median testosterone 5.7 nmol/L; median recovery times 9/13/18 months to 5/7.5/10 nmol/L; >90% recovered to thresholds.	Cessation defined as last injection +3 months; thresholds used instead of pre-ADT recovery; flash meds used in 56 patients.

Shah et al. [35] (2023)	Time to testosterone recovery (>230 ng/dL) and relationship to QOL recovery (EPIC-26)	At 12 months post-SBRT, 71% recovered to eugonadal testosterone with mean recovery time ~4 months; sexual/hormonal EPIC-26 scores improved in parallel and returned within MID by 12 months.	Pre-ADT testosterone not available for all; morning draws encouraged to reduce circadian variability.
Bandara et al. [36] (2019)	Fatigue trajectory and associations with testosterone/Hb/MRI marrow metrics	Fatigue increased significantly by 18 months; testosterone dropped to castrate levels by 3 months and remained low through 18 months, but changes in testosterone/Hb/MRI marrow metrics did not strongly associate with fatigue.	Small cohort; MRI marrow only baseline and 6 months; aims mechanistic fatigue modeling rather than RT toxicity endpoints.
Pilepich et al. [37] (1989)	Tumor clearance; testosterone suppression; LRC/DFS/OS; drug-related toxicity	DES produced greater median testosterone suppression than megestrol ($p<0.001$) but no difference in tumor clearance; DES associated with higher gynecomastia/fluid retention.	Hormones used as cytoreduction pre- and during RT; not modern LHRH-based ADT; eligibility excluded CV risk factors.
Berg et al. [38] (2009)	HRQoL (SF-36, HADS, Fatigue Questionnaire, BSFI) and correlation with sex hormones	Testosterone/EFT/estradiol/LH/FSH increased from baseline at 5 years, but hormone levels did not correlate with HRQoL; sexual domains markedly worse than controls.	Antiandrogen receptor blockade with maintained gonadal testosterone; cross-sectional HRQoL design; no RT-only control.
Nichols et al., RTOG 9408 [39] (2017)	Short-term testosterone change (end-RT and 3 months); subgroup WPRT vs PORT; attempted SAQ correlation (limited)	Median testosterone decline ~9% at RT completion and at 3 months post-RT; no significant difference between WPRT vs PORT.	Large missing post-RT testosterone due to decentralized collection; diurnal variation acknowledged.
Zagars and Pollack [40] (1997)	Endocrine kinetics; testicular scatter dose (TLD); PSA change	Mean testosterone decreased modestly at 3 months (~9% mean percentage fall); testicular scatter dose ~2.07 Gy for 68 Gy prostate dose.	Seasonal balance attempted; no longer follow-up; Leydig dose below injury threshold; paired nonparametric test.
Grigsby and Perez [41] (1986)	Endocrine kinetics; inferred testicular scatter dose (scrotal dosimetry)	Total testosterone remained unchanged up to 2 years; dihydrotestosterone decreased slightly; FSH and LH rose during RT and remained elevated up to 2 years, consistent with low-dose testicular irradiation.	Estimated scrotal scatter dose 5–8% of maximum (total 4.5–6.0 Gy testicular dose over 7–8 weeks).
Tomić et al. [42] (1983)	Endocrine effects vs absorbed gonadal dose; shielding effect	Testosterone decreased at 1 week and 3 months then returned by 6–12 months in paired cohort; LH/FSH increased (FSH rise from 1 week); higher gonadal dose (>10 Gy) associated with greater testosterone decrease; lead shielding reduced testicular dose ~50%.	Very high-energy betatron era; gonadal doses estimated from scrotal dosimetry; includes prolactin.
Pickles et al. [43] (2002)	Testosterone nadir/recovery; biochemical control (Vancouver + ASTRO); PSA doubling time in relapse	Median nadir at 6 months; testosterone fell to ~83% baseline; 7.5% had >50% decrease; 97% recovered to normal but only 60% to individual baseline; testosterone decline not associated with biochemical control or PSA doubling time.	No testicular shielding; sampling times varied by time-of-day; volumes associated with nadir.
Pompe et al. [44] (2017)	Testosterone decrease/recovery (to ≥90% BST) and association with biochemical recurrence (Phoenix)	75% had testosterone decrease (median time to first decrease 6.4 months; median nadir −30%); 45% developed biochemical hypogonadism (<8 nmol/L) and 63% recovered to ≥90% BST; no link to BCR.	Testosterone follow-up non-uniform across protocols; no QoL data; older age/BMI/higher BST predicted lower recovery.
Markovina et al. [45] (2014)	Testosterone kinetics; incidence of biochemical hypogonadism; correlation with estimated testicular dose	Small transient testosterone decrease at 6 months (mean −33 ng/dL), returning to baseline by 1 year; no increase in biochemical hypogonadism; testicular dose (incl imaging) not associated with change.	Testicular dose estimated (planning system or Kry method) plus imaging dose; majority testes out of field; erectile function data sparse.

Ishiyama et al. [46] (2012)	Endocrine kinetics; correlation with mean testicular dose; PSA failure	Testosterone declined over time; significant decreases at 12, 24, 30, 36 m; dose–ratio correlation significant only at 4 m.	High baseline hypogonadism (66%); testis contouring may include adjacent structures.
Williams et al. [47] (2019)	Long-term testosterone kinetics; technique/energy/field size effects	No difference in testosterone decline rates between EBRT groups and brachytherapy-only controls over ~5 years; EBRT technique/energy/field size not associated with decline rate.	Adjusted for BMI/diabetes/opioids/prostatectomy; time-of-day draws variable; daily portal imaging.
Usera et al. [48] (2020)	PFS/MFS/OS by baseline testosterone strata; testosterone recovery/trends post-therapy in no-ADT subgroup	Baseline testosterone (>100 ng/dL) not associated with PFS/MFS/OS after propensity adjustment; in men not using ADT, no significant change in testosterone at 1 year regardless of therapy type.	Baseline cohort stratified by 1 SD around mean; labs decentralized but assay specified; separate no-ADT subgroup for post-treatment kinetics.
Oermann et al. [49] (2011)	Testosterone change; biochemical/clinical hypogonadism; QoL and toxicity; testicular scatter dose	Median testosterone fell ~24% at 1 year (testicular scatter median 2.1 Gy) but biochemical hypogonadism rate unchanged and EPIC sexual/hormonal scores not significantly changed.	Beams avoiding direct testis traversal; defines biochemical hypogonadism <8 nmol/L; longer follow-up needed for recovery.
Yuan et al. [50] (2019)	Testosterone percent change and sexual/hormonal QoL changes post-SBRT	Transient small testosterone decline early after SBRT that normalized by 24 months; no clinically meaningful decline in EPIC hormonal domain; small-sized effect in EPIC sexual domain at 19–24 months.	Analyses stratified by baseline testosterone tertiles; mixed-effects models used; no testicular dose data.
Taniguchi et al. [51] (2019)	Testosterone kinetics; biochemical hypogonadism (STL <3.0 ng/mL); predictors of decline	STL declined significantly by 3–6 months (median nadir 89.4% at 6 months) and recovered to baseline by 18 months; baseline STL predicted hypogonadism risk.	STL measured at variable times of day; baseline hypogonadism present in 12.7% and excluded from predictor models.
Nichols et al. [52] (2012)	Testosterone change vs baseline; subgroup by age, dose, ethnicity	No significant change in median testosterone at completion or at 6–24 months after proton therapy.	Follow-up testosterone availability declined over time due to insurance coverage; Wilcoxon signed-rank tests used; no pelvic nodes; one field daily lateral/lateral-oblique.
Kil et al. [53] (2013)	Testosterone change vs baseline; subgroup by dose/target volume/age	No statistically significant testosterone change at completion, 6 months, or 12 months after hypofractionated proton therapy.	Paired Wilcoxon; daily online kV imaging; analysis limited by missing follow-up testosterone in some patients.
Planas et al. [54] (2016)	Hormone axis changes comparing EBRT vs RP; testicular scatter dose estimate	EBRT associated with higher LH/FSH and lower total/free testosterone at 3 months, with persistent lower testosterone and elevated FSH at 12 months vs RP.	Estimated mean testicular scatter dose 0.47 Gy; groups differed in age/risk; retrospective analysis.
Hattori et al. [55] (2021)	Longitudinal testosterone and sexual QoL after IGPT with/without CAB by risk group	Low-risk IGPT: testosterone did not decrease but sexual function scores declined over time; CAB groups: testosterone and PSA rose after CAB termination with partial recovery of EHS/EPIC sexual scores (not exceeding MID).	Testosterone measured only up to 3 years after IGPT due to insurance; excluded biochemical failures ≥36 months; cultural baseline sexual scores low.
**IL-6 / inflammation domain**			
Stanojković et al. [12] (2020)	Acute GU toxicity (RTOG/EORTC modified by Peeters); lymphocyte subsets; cytokine gene expression	Increased serum IL-6 during RT independently associated with higher grade acute GU toxicity.	Definitive vs post-op differed in IL-6 and IFN-γ levels; prospective sampling at six timepoints.
Lopes and Callera [11] (2012)	Cytokine kinetics during RT; PSA response	IL-6 rose significantly at day 15 then declined to pre-RT levels by day 30; other cytokines unchanged.	Majority received concurrent hormone therapy but subgroup analysis reported similar IL-6 pattern.

Holliday et al. [56] (2016)	Fatigue (Actiwatch Score), sleep metrics (actigraphy), PROs (SF-36, IPSS); cytokine trajectories	Fatigue increased during EBRT; IL-6 showed a non-significant upward trend (P=0.09) and cytokine levels were not significantly associated with fatigue trajectories.	High temporal precision (1 h post-fx) to capture cytokine peaks; excluded ADT/depression to reduce confounding.
Johnke et al. [57] (2009)	Cytokine kinetics during RT; comparison RT-only vs TAS+RT (immunologic impact)	IL-1β and IL-6 rose early and peaked ~1–2 weeks into RT; TAS increased magnitude of IL-1β/IL-6 and reduced TGFβ compared with RT alone; TGFβ showed two waves (1–2 weeks and 5–6 weeks).	Highly granular dose-anchored sampling; designed for immunologic profiling rather than clinical toxicity linkage.
Singh et al. [58] (2020)	Cytokine kinetics; exploratory association with treatment effects (no formal toxicity linkage)	Post-ADT: TGF-β1 ↑ and IL-6 ↓ (NS); post-RT: TGF-β1 ↑ (NS); at 3 months post-RT: IL-8 ↑ significantly, IL-6 ↑ and TGF-β1 ↓.	Small sample; non-significant changes except IL-8 at 3 months; plasma processing standardized and blinded.
Christiansen et al. [59] (2007)	Acute GI toxicity (CTC proctitis); hematologic changes (Hb/WBC); biomarker dynamics	Patients developing acute proctitis had significant increases in plasma pro-hepcidin, IL-6 and TNF-α with concurrent Hb decrease; no proctitis group showed different iron-marker changes.	IL-1β below detection throughout; urine pro-hepcidin available only in subset; 'maximum during RT' used for comparisons.
Pinkawa et al. [60] (2015)	Acute/late urinary & bowel toxicity via EPIC changes; predictive value of early hematologic/cytokine changes	Early changes at 2–3 weeks (T2), especially CRP elevation and Hb drop, predicted acute urinary toxicity; lymphocyte response and IL-6 status at T2 associated with late toxicity patterns.	IL-6 often below detection (<1.5 ng/L); authors analyzed proportions above thresholds rather than continuous change.
Kopčalić et al. [61] (2022)	Associations of IL-6/TGF-β1 with acute GU/GI toxicity and fatigue; longitudinal toxicity follow-up	IL-6 increased significantly by fraction 25; pretreatment IL-6 correlated with maximum fatigue; pretreatment and on-treatment TGF-β1 associated with acute GU toxicity.	Defines baseline symptoms at fraction 5 due to low dose; mixed RT techniques; small sample and limited late-tox window.
Bedini et al. [62] (2018)	Inflammatory mediator kinetics vs dose/time; association with acute/late rectal toxicity; effect of prior abdominal surgery	CCL2 increased significantly during IMRT and remained elevated at end; patients with grade 2 late rectal toxicity had end-RT CCL2 above median; IL-1β/IL-6 largely undetectable.	Pilot exploratory; late symptoms defined as max grade 6 months–5 years; PROM-based SOMA/LENT mapping.
Christensen et al. [63] (2009)	Acute GI/GU toxicity vs cytokine trajectories; feasibility of longitudinal proteomics	IL-6 and IFN-γ increased significantly during IMRT; IL-2 change associated with acute GI toxicity and IL-1 with acute GU toxicity; IL-6 peak predicted around ~42 Gy (quadratic dose relation).	High interpatient variability; serum vs plasma correlated but urine not; exploratory pilot cohort.
Hallemeier et al. [64] (2013)	GI enteritis (CTCAE v3), bowel movements/cramping; cytokine kinetics; DVH correlation	No significant change in mean IL-6 or TGF-β levels across baseline, week 3, and final week; no correlation with acute enteritis or bowel DVH.	Small mixed-disease cohort; timing not fraction-anchored; 1 missing lab per timepoint; one spurious excluded.
Joensuu et al. [65] (2010)	Safety/DLTs; PSA relapse-free survival; correlative cytokines vs ALT elevation	Serum TNF/IL-1α/IL-6 levels were generally low and did not correlate with ALT elevation during gefitinib+RT.	Gefitinib-related transaminitis common; Cytokine sampling timepoints were not clearly specified, limiting interpretation of the inflammatory biomarker findings.
**γ-H2AX / DDR domain**			
Zwicker et al. [66] (2011)	In vivo dose distribution in lymphocytes (DLH) and comparison with DVH; technique differences	SSIMRT produced more low-dose–exposed lymphocytes (<20%) and fewer mid-dose (30–60%) vs 3D; method feasible for in vivo dosimetry.	Circulating lymphocytes may be irradiated multiple times during longer beam-on time; absolute Gy inference limited.
Zwicker et al. [67] (2015)	In vivo biodosimetry (dose–lymphocyte histogram); technique comparison TOMO vs SSIMRT	TOMO: fewer low-dose–exposed lymphocytes (<40% prescribed) and lower mean γ-H2AX foci/lymphocyte vs SSIMRT.	Ex vivo calibration line; background foci subtracted; fixation standardized 2 h post-fx.
Zahnreich et al. [68] (2015)	Biodosimetry: estimate equivalent whole-body dose and partial-body dose; dose distribution in leukocytes;	γ-H2AX foci 30 min post-fraction 1 correlated linearly with equivalent whole-body dose in prostate RT and provided rapid dose estimates;	Standardized cold handling to block repair; extensive calibration curves; includes non-prostate cohorts; prostate subgroup treated with RapidArc.

	comparison of γ-H2AX vs cytogenetics	residual foci at 24 h better reflected local high-dose exposure; cytogenetics less reliable immediately.	
El-Saghire et al. [69] (2014)	Systemic low-dose (out-of-field) biological response: DNA damage and immune/inflammatory gene expression signatures	All patients showed γ-H2AX induction 30 min after fraction 1 with persistent DDR gene expression at 18–24 h, alongside immune/inflammatory signaling activation in whole blood.	Equivalent total body blood dose per fraction ~31±8 mGy estimated from planning; highlights systemic low-dose effects.
Osman et al. [70] (2017)	Dose-dependent DNA damage kinetics; correlations with acute/late toxicity (EPIC) and post-implant dosimetry (D90, rectum/bladder D0.1cc/D2cc; sector analysis)	Higher γH2AX/53BP1 induction at 24h (relative to baseline) correlated with late bowel symptoms at 1–2 years; dosimetry metrics alone correlated poorly with late toxicity.	Small sample; exploratory correlations without multiplicity correction; persistent foci increase up to 4 weeks despite source decay suggests prolonged DDR/inflammatory effects.

Abbreviations: bDFS, biochemical disease-free survival; BCR, biochemical recurrence; BFI, Brief Fatigue Inventory; BSFI, Brief Sexual Function Inventory; CTCAE, Common Terminology Criteria for Adverse Events; DFS, disease-free survival; DVH, dose-volume histogram; EPIC, Expanded Prostate Cancer Index Composite; EORTC, European Organisation for Research and Treatment of Cancer; GI, gastrointestinal; GU, genitourinary; HADS, Hospital Anxiety and Depression Scale; IIEF-5, International Index of Erectile Function-5; IPSS, International Prostate Symptom Score; MFS, metastasis-free survival; OS, overall survival; PCSS, prostate cancer-specific survival; PROMs, patient-reported outcome measures; PROMIS, Patient-Reported Outcomes Measurement Information System; QoL, quality of life; RTOG, Radiation Therapy Oncology Group; SHIM, Sexual Health Inventory for Men; SOMA/LENT, Subjective-Objective-Management-Analytic / Late Effects of Normal Tissues.

Implementation note: Practical observations were restricted to workflow, sampling, processing, assay, and follow-up issues reported in the included studies or directly evident from their study designs. Formal cost, cost-effectiveness, turnaround-time, personnel-resource, and implementation-readiness analyses were not reported in the mapped evidence.
