# Supplementary material for: Selected Blood-Accessible Biomarkers in Prostate Cancer Radiotherapy: A PRISMA-ScR Timing-Window Framework Beyond PSA
Source: Cancers (Basel). 2026 Jul 25;18(15):2398. doi: 10.3390/cancers18152398 (PMC13463946; doi:10.3390/cancers18152398)
Supplement: Supplementary file 1 [file cancers-18-02398-s001.zip › Supplementary Table S2.pdf]

Supplementary Table S2. Cross-domain methodological reporting and implementation overview of included studies.

Domain	Total studies	RT-only	RT+ADT/CAB	RT+Other systemic	Mixed/comparator-defined	Assay platform specified (n)	Sampling schedule detailed (n)	Pelvic nodal RT status classifiable (n)	PROMs/symptom instruments (n)	Feasibility/implementation note
**Testosterone**	28	14	11	1	2	15	28	20	8	Generally compatible with routine serum laboratory workflows, but longitudinal testing creates cumulative follow-up and resource requirements. Interpretation requires standardized collection timing, consideration of diurnal variation, harmonized recovery thresholds, and explicit anchoring to RT or ADT cessation. Formal cost data were not reported.
**IL-6 / inflammation**	12	4	1	1	6	11	11	5	4	Requires standardized specimen matrix, collection timing, processing interval, storage conditions, and assay platform. Low circulating concentrations, platform-specific detection limits, batch effects, multiplex quality-control requirements, and repeated during-RT sampling may increase laboratory and coordination burden. Formal cost data were not reported.
**γ-H2AX / DDR**	5	4	0	0	1	5	5	4	1	Requires tightly timed post-fraction blood collection, controlled processing intervals, immunofluorescence staining, microscopy, and foci scoring. Workflow, staffing, and coordination requirements may be substantial; centralized or automated analysis may improve

										scalability but requires technical validation. Formal cost data were not reported.
**Galectins**	0	0	0	0	0	0	0	0	0	No eligible RT-anchored circulating studies were identified. Circulating detectability, assay reproducibility, sampling feasibility, workflow requirements, clinically relevant thresholds, and costs therefore remain undefined.

Abbreviations: ADT, androgen deprivation therapy; CAB, combined androgen blockade; DDR, DNA damage response; PROMs, patient-reported outcome measures; RT, radiotherapy.

Note: Counts were derived from Supplementary Table S1 and reflect study-level methodological reporting rather than risk of bias or study quality. "Mixed/comparator-defined" includes studies with mixed treatment contexts, mixed disease cohorts, or explicit subgroup/comparator structures that were not reducible to a single RT-context category. "Assay platform explicitly specified" indicates that a recognizable analytical method or platform was reported (for example ELISA, radioimmunoassay, chemiluminescent immunoassay, multiplex assay, or immunofluorescence foci scoring), rather than being left as not reported or routine laboratory testing without assay detail. "Sampling schedule explicitly detailed" indicates that the paper reported more than a generic baseline/follow-up statement and provided interpretable timing anchors. "Pelvic nodal RT status explicitly classifiable" indicates that pelvic nodal irradiation could be coded from the study report as treated, not treated, or mixed/variable, rather than not reported. "PROMs/symptom instruments included" refers to validated patient-reported outcome measures or structured symptom instruments reported in the study. Feasibility and implementation observations are qualitative and were derived from the mapped sampling, assay, processing, and follow-up characteristics; they do not constitute a formal economic evaluation or comparative assessment of implementation readiness.
